# Supplementary material for: Long non-coding RNAs are essential for Schistosoma mansoni pairing-dependent adult worm homeostasis and fertility
Source: PLoS Pathog. 2023 May 5;19(5):e1011369. doi: 10.1371/journal.ppat.1011369 (PMC10191282; doi:10.1371/journal.ppat.1011369)
Supplement: S1 Text — (PDF) [file ppat.1011369.s001.pdf]

S1 Text

Long non-coding RNAs are essential for *Schistosoma mansoni* pairing-dependent adult worm homeostasis and fertility

Gilbert O. Silveira[1,2]; Helena S. Coelho[1]; Adriana S. A. Pereira[1,2]; Patrícia A. Miyasato[3]; Daisy W. Santos[1,2]; Lucas F. Maciel[1]; Giovanna G. G. Olberg[1]; Ana C. Tahira[1]; Eliana Nakano[3]; Maria Leonor S. Oliveira[4]; Murilo S. Amaral[1*]; Sergio Verjovski-Almeida[1,2*]

[1]Laboratório de Ciclo Celular, Instituto Butantan, 05503-900 São Paulo, SP, Brazil

[2]Instituto de Química, Universidade de São Paulo, 05508-900 São Paulo, SP, Brazil

[3]Laboratório de Parasitologia, Instituto Butantan, 05503-900 São Paulo, SP, Brazil

[4]Laboratório de Bacteriologia, Instituto Butantan, São Paulo 05503-900, SP, Brazil

*Corresponding author: Murilo S. Amaral – murilo.amaral@butantan.gov.br

*Corresponding author: Sergio Verjovski-Almeida – sergio.verjovski@butantan.gov.br

1. Supplementary Data ……………………………………………page 2

2. Supplementary Methods …………………………………….. page 11

3. Figs A to H ……………………………………………………….. page 19

1. Supplementary Data

To check if the *in vitro* mimetic model has characteristics similar to the mixed-sex/single-sex *in vivo* infection model used by Lu et al., 2016 [1], we performed RT-qPCR assays to measure protein-coding genes known to be differentially expressed (DE) in the mixed-sex/single-sex *in vivo* infection model. We first conducted a screening on genes that could be used as reference genes for the RT-qPCR analyses, by looking at the genes whose expression was more stable in the re-analysis of: (1) all RNA-Seq libraries from Lu et al., 2016 [1], (2) all adult worm RNA-Seq libraries from Lu et al., 2016 [1] and (3) the three best reference genes found in a previous analysis in the literature [2]. The efficiencies of RT-qPCR primers for all candidate reference genes are shown in **Table D in S1 Appendix.** The Cq values obtained in the RT-qPCR analyses of the putative candidate reference genes measured in all paired and unpaired male and female *in vitro* cultured samples are shown in **Table J in S1 Appendix**. A gene expression stability survey from all candidate reference genes was taken with GeNorm [3] **(Table K in S1 Appendix)** and NormFinder [4] **(Table L in S1 Appendix)**. Our analysis found Smp_099690 (*Protein RER1*) and Smp_023150 (*Serine/threonine-protein phosphatase 6 catalytic subunit*) as the two most stable genes in our 14 different samples from adult worms cultured *in vitro* for up to 8 days either paired or not.

Then, we measured by RT-qPCR the expression of 14 protein-coding genes as controls, including genes that are differentially expressed between females and males (see next paragraph). The efficiencies of RT-qPCR primers for all protein-coding genes used are shown in **Table E in S1 Appendix,** and the Cq values obtained from this analysis are shown in **Table M in S1 Appendix.**

In **Fig I, panel A**, we show the expression of the p14 gene (Smp_316140) in males and females cultured *in vitro* either paired or unpaired and compared the expression with the data retrieved from our re-analysis of the RNA-Seq from Lu et al., 2016 [1].

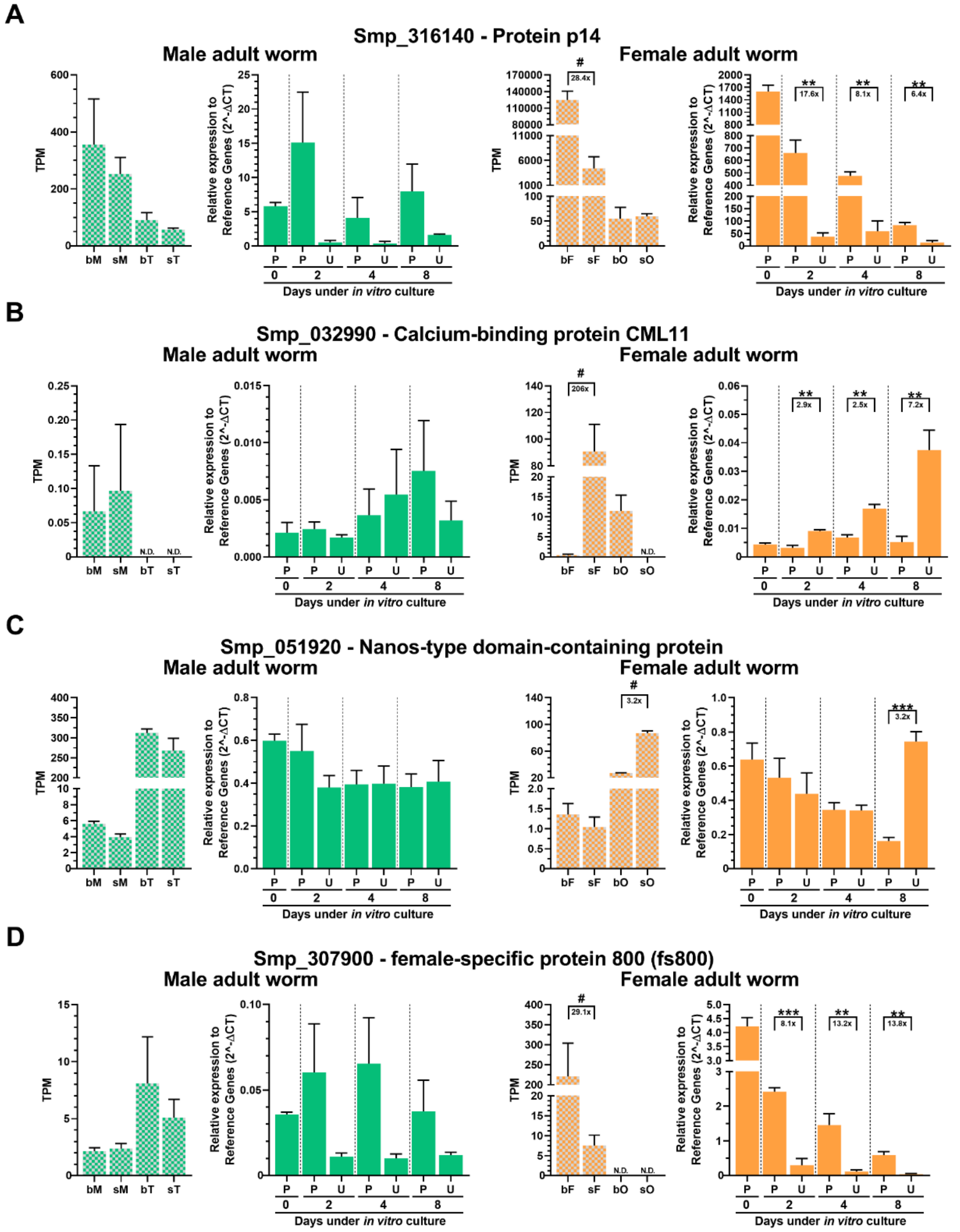

Fig I. Expression validation of protein-coding genes known to be related to the reproductive system and measured by RT-qPCR in *S. mansoni* cultured *in* vitro for 2, 4 or 8 days as paired couples or unpaired males and females. Four protein-coding genes were selected for RT-qPCR assays, namely (**A**) Smp_316140, Protein p14; (**B**) Smp_032990, Calcium binding protein CML11; (**C**) Smp_051920, Nanos type domain-containing protein; and (**D**) Smp_307900, female specific protein 800 (fs800). Male related results are shown on the left (green) and female results on the right (orange). Paired couples (P) or unpaired (U) parasites were obtained by perfusion of hamsters infected for 42 days with *S. mansoni* cercariae. After perfusion, males and females were

cultured *in vitro* for 2, 4 or 8 days as paired (P) couples or unpaired (U) worms. RT-qPCR results (solid-colored graphs) are normalized to the geometric mean of reference genes Smp_099690 and Smp_023150. Expression values from 4 different biological replicates are shown. Standard error of the mean (SEM) is shown in the error bars. (**) = $p < 0.01$; (***) = $p < 0.001$, Student t test. N.D.: Not detected. ns: p-value > 0.05. For comparison, RNA-Seq data from re-analysis of Lu et al., 2016 [1] is shown (plaid-colored graphs) and the expression is measured in TPM (transcripts per million); RNA-Seq data is retrieved from males (M), females (F), testes (T) or ovaries (O) from either a mixed-sex (b) or a single-sex (s) infection; (#) = FDR<0.005. The fold-change differences between the compared groups are represented under the brackets.

Expression of the p14 gene was mainly present in females when compared with males, and mostly present in females obtained from mixed-sex infection. Our *in vitro* mimetic model analysis has shown that the p14 gene was differentially expressed when comparing paired and unpaired females *in vitro* cultured for 2, 4, and 8 days. Notably, even though the ABC medium was developed to adequately sustain females egg deposition *in vitro*, the p14 gene expression was sensitive to *in vitro* culturing of the parasites **(Fig I, panel A)**.

On the other hand, when looking at the expression pattern of *Calcium-binding protein CML11* gene (Smp_032990) **(Fig I, panel B)**, another gene mostly expressed in females, we have observed an *in vitro* culturing time-dependent differential expression in unpaired females when compared with paired females. When looking at the Smp_051920 *Nanos-type domain-containing protein* **(Fig I, panel C)**, a 3.2-fold higher expression in unpaired females was observed when compared to paired females cultured *in vitro* for 8 days; this is in line with its higher expression in the male and female gonads when compared to the whole worm in the re-analysis of Lu et al., 2016 [1] data, and with the significantly higher expression in the ovaries retrieved from single-sex infections compared with mixed-sex infections **(Fig I, panel C)**. Further, another gene that is mostly expressed in females has been measured, the female-specific protein 800 (fs800, Smp_307900), and the differential expression was increased in culture in a time-

dependent manner in the paired females when compared with the unpaired ones **(Fig I, panel D)**.

Other genes such as *Egg Shell Protein* (Smp_000430), *Nucleotide pyrophosphatase: phosphodiesterase 5* (Smp_153390), *Potassium channel toxin gamma-KTx* (Smp_194830), *Heat shock 70 kDa protein homolog* (Smp_302170), and *Tumor necrosis factor receptor superfamily member 16* (Smp_332480) **(Fig J)** have been validated in our *in vitro* mimetic model when comparing with the data retrieved from the re-analysis of Lu et al, 2016 [1] data.

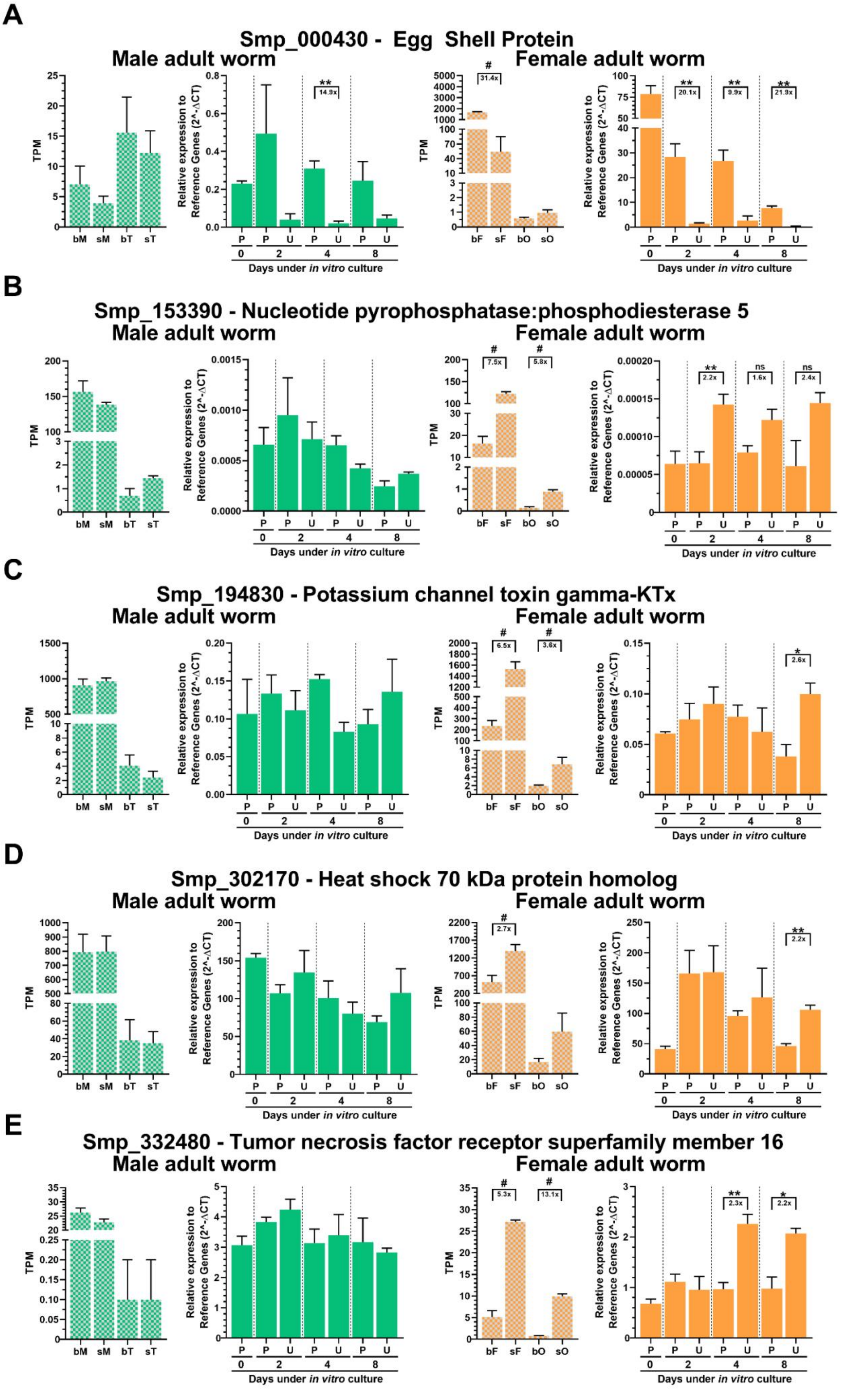

Fig J. Expression validation of protein-coding genes known to be related to the reproductive system and measured by RT-qPCR in *S. mansoni* cultured *in* vitro for

2, 4 or 8 days as paired couples or unpaired males and females. Four protein-coding genes were selected for RT-qPCR assays, namely (**A**) Smp_000430, Eggshel protein; (**B**) Smp_153390, Nucleotide pyrophosphatase:phosphodiesterase 5; (**C**) Smp_194830, Potassium channel toxin gamma-KTx; (**D**) Smp_302170, Heat shock 70 kDa protein homolog; and (**E**) Smp_332480, Tumor necrosis factor receptor superfamily member 16. Male related results are shown on the left (green) and female results on the right (orange). Paired couples (P) or unpaired (U) parasites were obtained by perfusion of hamsters infected for 42 days with *S. mansoni* cercariae. After perfusion, males and females were cultured *in vitro* for 2, 4 or 8 days as paired (P) couples or unpaired (U) worms. RT-qPCR results (solid-colored graphs) are normalized to the geometric mean of reference genes Smp_099690 and Smp_023150. Expression values from 4 different biological replicates are shown. Standard error of the mean (SEM) is shown in the error bars. (*) = $p < 0.05$; (**) = $p < 0.01$, Student t test. N.D.: Not detected. ns: p-value > 0.05. For comparison, RNA-Seq data from re-analysis of Lu et al., 2016 [1] is shown (plaid-colored graphs) and the expression is measured in TPM (transcripts per million); RNA-Seq data is retrieved from males (M), females (F), testes (T) or ovaries (O) from either a mixed-sex (b) or a single-sex (s) infection; (#) = FDR<0.005. The fold-change differences between the compared groups are represented under the brackets.

When looking at the *Calcium release-activated calcium channel protein 1* (Smp_076650), *Neurocalcin homolog* (Smp_085650), and the *Vasa-like DEAD-box RNA helicase* genes (Smp_068440), their expression profiles in the Lu et al. [1] model and our mimetic model could suggest a tendency for validation, but no significant differential expression was seen **(Fig K)**.

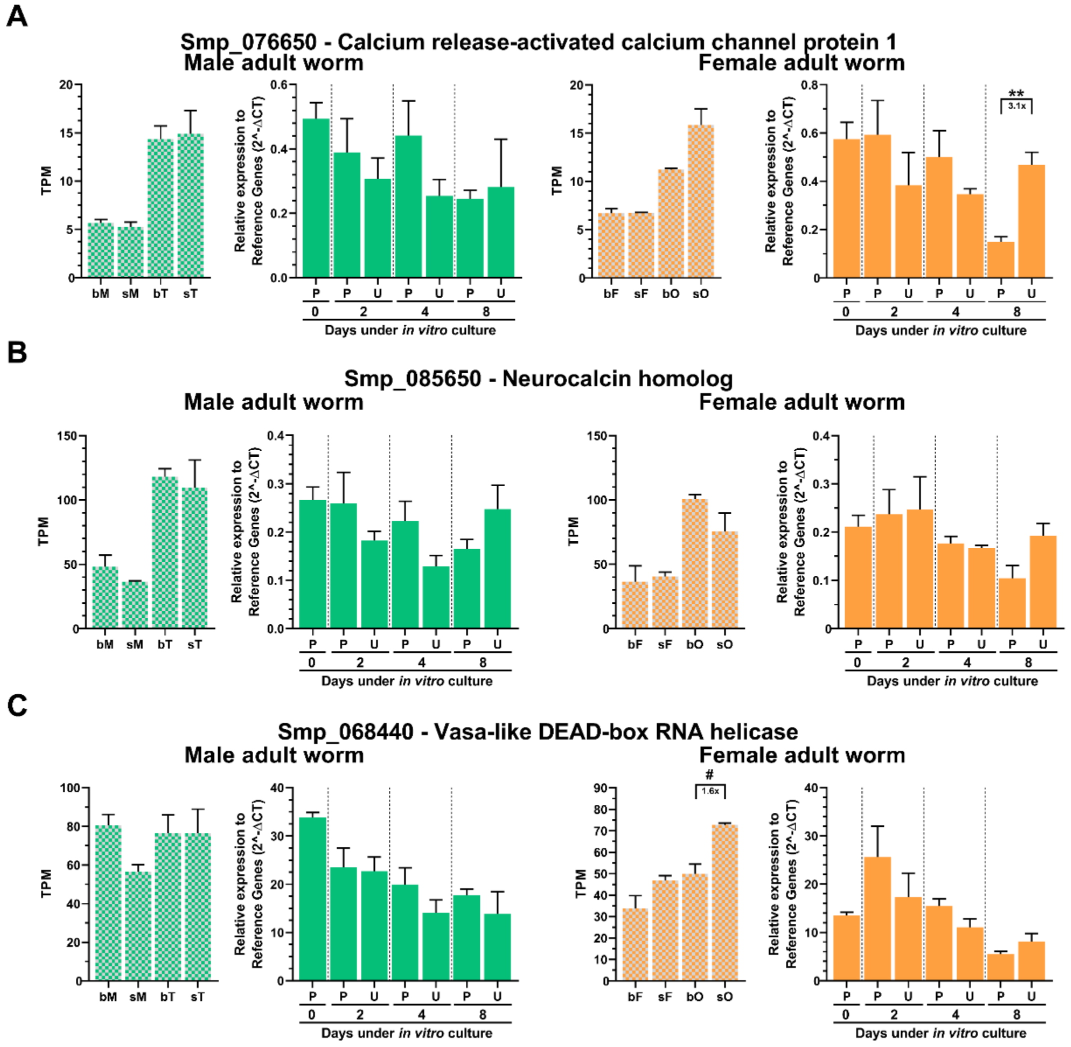

Fig K. Expression validation of protein-coding genes known to be related to the reproductive system and measured by RT-qPCR in *S. mansoni* cultured *in* vitro for 2, 4 or 8 days as paired couples or unpaired males and females. Three protein-coding genes selected for RT-qPCR assays were validated as non-differentially expressed genes in the *in vitro* mimetic assays, namely (**A**) Smp_076650, Calcium release-activated calcium channel protein 1; (**B**) Smp_085650, Neurocalcin homolog; and (**C**) Smp_068440, Vasa-like DEAD-box RNA helicase. Male related results are shown on the left (green) and female results on the right (orange). Paired couples (P) or unpaired (U) parasites were obtained by perfusion of hamsters infected for 42 days with *S. mansoni* cercariae. After perfusion, males and females were cultured *in vitro* for 2, 4 or 8 days as paired (P) couples or unpaired (U) worms. RT-qPCR results (solid-colored graphs) are normalized to the geometric mean of reference genes Smp_099690 and Smp_023150. Expression values from 4 different biological replicates are shown. Standard error of the mean (SEM) is shown in the error bars. (**) = $p < 0.01$, Student t test. For comparison, RNA-Seq data from re-analysis of Lu et al., 2016 [1] is shown (plaid-colored graphs) and the expression is measured in TPM (transcripts per million); RNA-Seq data is retrieved from males (M), females (F), testes (T) or ovaries (O) from either a mixed-sex (b) or a

single-sex (s) infection; (#) = FDR<0.005. The fold-change differences between the compared groups are represented under the brackets.

Finally, the *Putative Nanos RNA binding protein* (Smp_055740) and the *5-hydroxytryptamine receptor 1A* (Smp_126730) were two genes not validated when comparing the mixed-sex/single-sex cercariae infection model with our *in vitro* culturing model **(Fig L)**.

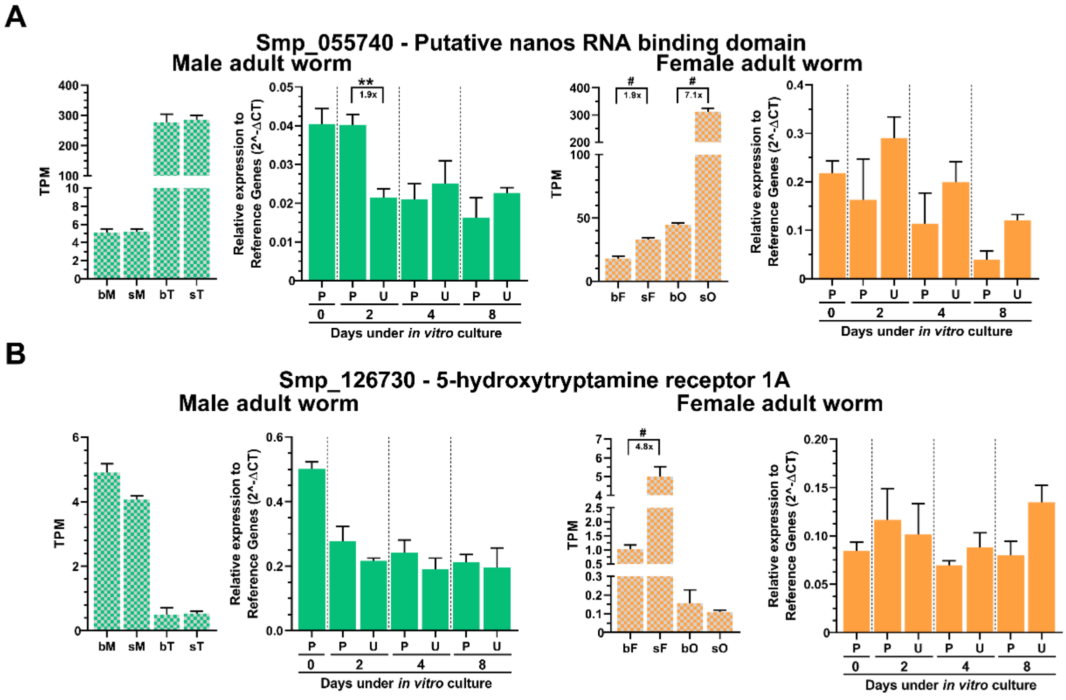

Fig L. Expression validation of protein-coding genes known to be related to the reproductive system and measured by RT-qPCR in *S. mansoni* cultured *in* vitro for 2, 4 or 8 days as paired couples or unpaired males and females. Two selected protein-coding genes were not validated by RT-qPCR in the *in vitro* mimetic assays, namely (**A**) Smp_055740, Putative nanos RNA binding domain; and (**B**) Smp_126730, 5-hydroxytryptamine receptor 1A. Male related results are shown on the left (green) and female results on the right (orange). Paired couples (P) or unpaired (U) parasites were obtained by perfusion of hamsters infected for 42 days with *S. mansoni* cercariae. After perfusion, males and females were cultured *in vitro* for 2, 4 or 8 days as paired (P) couples or unpaired (U) worms. RT-qPCR results (solid-colored graphs) are normalized to the geometric mean of reference genes Smp_099690 and Smp_023150. Expression values from 4 different biological replicates are shown. Standard error of the mean (SEM) is shown in the error bars. (**) = p < 0.01, Student t test. For comparison, RNA-Seq data from re-analysis of Lu et al., 2016 [1] is shown (plaid-colored graphs) and the expression

is measured in TPM (transcripts per million); RNA-Seq data is retrieved from males (M), females (F), testes (T) or ovaries (O) from either a mixed-sex (b) or a single-sex (s) infection; (#) = FDR<0.005. The fold-change differences between the compared groups are represented under the brackets.

2. Supplementary Methods

2.1 Selection of lincRNAs to be tested for their involvement in adult worm pairing

2.1.1 Pattern of lncRNAs expression in immature and mature males and females

Because our RNA-Seq re-analysis used the reference transcriptome of Maciel et al., 2019 [5] comprised of lncRNAs besides protein-coding RNAs, and 16,583 lncRNAs were detected as expressed in the *S. mansoni* samples in addition to 14,520 protein-coding genes, we first performed an overall inspection of the patterns of expression, using a principal components analysis (PCA) of the entire set of expressed lncRNAs and protein-coding RNAs. A pattern similar to that of Lu et al., 2016 [1] was obtained **(Fig M)**; of note, the expression pattern of immature single-sex whole females was clustered closer to that of single-sex and mixed-sex whole males, whereas the mixed-sex female samples clustered quite apart **(Fig M)**, similar to what had been shown by Lu et al., 2016 [1] for the pattern of protein-coding genes alone. This suggests a pattern of lncRNAs expression in the tissues and organs of immature and mature males and females that closely follows the patterns of expression of protein-coding genes.

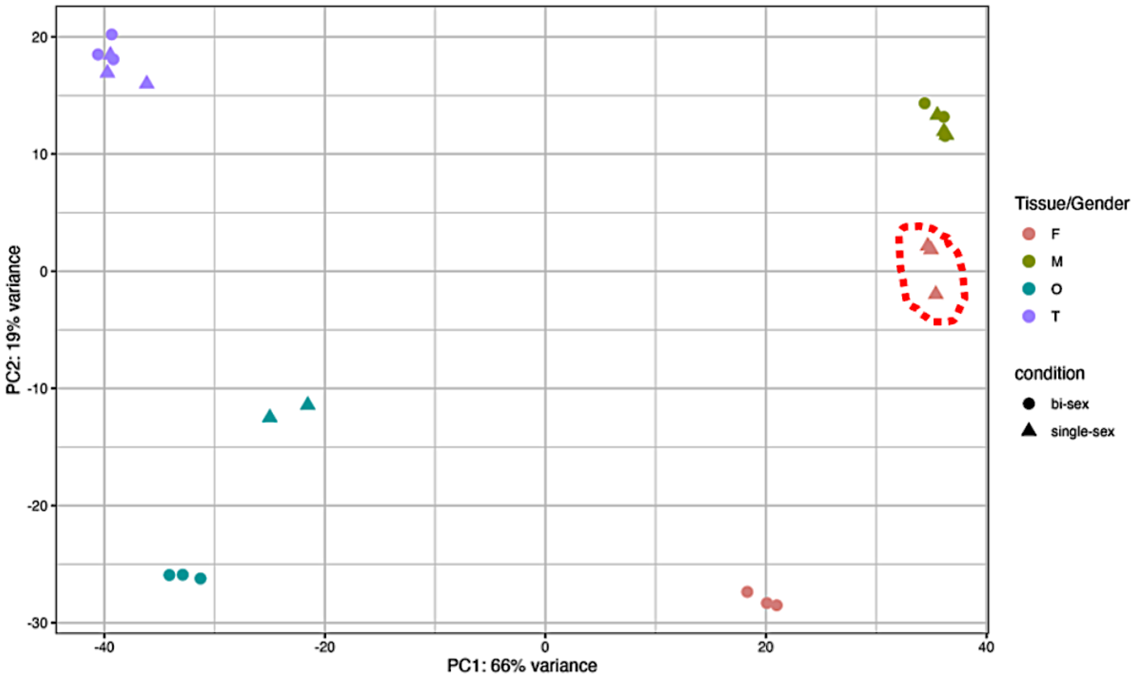

Fig M. Principal Component Analysis of expression profiles of *S. mansoni* parasites and their gonads. Principal component analysis (PCA) of expressed genes (dots and triangles) in three biological replicates (different dots or triangles) of *S. mansoni* parasites retrieved from single-sex (triangles) and mixed-sex infections (dots). Females from mixed-sex infections are represented by the orange dots, and from single-sex infections, by the orange triangles. Males from mixed-sex infections are represented by the green dots, and from single-sex infections by the green triangles. Ovaries from mixed-sex infection females are represented by the blue dots, and from single-sex infection females by the blue triangles. Testes from mixed-sex infection males are represented by the purple dots, and from single-sex infection males by the purple triangles. Variance stabilizing transformation (VST) from counts data was used to generate the PCA plot. Single-sex female samples are highlighted by the red dashed line.

2.1.2 Identification of lincRNAs enriched in mixed-sex females, males, and their reproductive organs

To identify a subset of lincRNA candidates to be tested for their possible involvement in adult worm pairing, we devised the filtering pipeline described below to single-out the lincRNAs enriched in mixed-sex females, males, and their reproductive organs.

First, we took each of the mixed-sex female, male, ovary, and testes samples and performed the most relevant pairwise comparisons between samples (**Table N)**, computing the number of significant DE transcripts detected in each comparison. The

lists of all DE transcripts found in each comparison are shown in **Tables O to X in S2 Appendix**.

Table N. Number of differentially expressed (DE) transcripts at each of the *Schistosoma mansoni* pairing status comparisons analyzed[a].

Analyzed Conditions	Total number of DE transcripts	Number of DE Smps	Number of DE lncRNAs	Number of DE lincRNAs	LincRNAs upregulated in the comparison	LincRNAs downregulated in the comparison
bF x sF	5070	4482	588	275	175	100
bF x bM	5264	4638	626	263	172	91
bF x bO	6797	5860	937	430	256	174
bM x sM	718	667	51	25	12	13
bM x bT	6912	5814	1098	473	196	277
bO x bT	6532	5563	969	409	155	254
bO x sO	5990	5232	758	347	183	164
bO x bM	9102	7426	1676	775	364	411
bT x sT	77	63	14	5	3	2
bT x bF	5922	5068	854	379	233	146

[a] The results presented here are based on a re-analysis of Lu et al., 2016 [1] data, which now also includes long non-coding RNAs besides protein-coding genes. Statistically significant DE genes were determined with DESeq2 (FDR < 0.05). Smps: *S. mansoni* protein-coding transcripts. lncRNAs: long non-coding RNAs. lincRNAs: long intergenic non-coding RNAs, a subgroup of the lncRNAs. LincRNAs upregulated in the comparison: number of lincRNAs that were more expressed in the first analyzed condition compared to the second one. LincRNAs downregulated in the comparison: number of lincRNAs that were more expressed in the second analyzed condition compared to the first.

The subgroup of DE long intergenic ncRNAs (DE lincRNAs), which is contained within the group of DE lncRNAs, was singled out in a separate column, as we concentrated for further analyses on those lincRNAs that were upregulated in each of the indicated comparisons (**Table N)**.

The intergenic lncRNAs (lincRNAs) were chosen because interfering with their expression levels with knockdown approaches (such as using double-stranded RNAs) does not suffer from the problem of a simultaneous artifactual knockdown of a protein-

coding message from the same locus. In fact, targeting a sense or an antisense lncRNA with long double-stranded RNAs (dsRNAs) may unwantedly reduce the expression of the protein-coding gene that is expressed from the locus, because the long dsRNA will share a stretch of its sequence with an intron of the immature pre-mRNA of the protein-coding gene message transcribed in the locus. An intergenic lncRNA is, by definition, away from the locus of a protein-coding gene, thus it will not share any stretch of sequence with the pre-mRNA message of a protein-coding gene.

2.1.3 A subset of lncRNAs show higher expression in mixed-sex worms or gonads compared with single-sex worms

Next, to further focus on a reduced list of differentially expressed lincRNAs that could play roles in *S. mansoni* worm sexual development and pairing, we excluded the lincRNAs with TPM values lower than 2 (lowly expressed), and cross-referenced the lists of lincRNAs found as significantly more expressed in mixed-sex females (bF) compared with single-sex females (sF), with mixed-sex ovaries (bO) and with mixed-sex males (bM), and found 31 bF enriched lincRNAs in common among these three pairwise comparisons **(Fig N, red circle).**

Similarly, when looking at lincRNAs significantly more expressed in mixed-sex males (bM), we found four bM lincRNAs in common in comparison with sM, bT, and bF, with an additional 44 lincRNAs in common in comparison with bT and bF alone, and 4 lincRNAs significantly more expressed in bM than in sM **(Fig O, red circles)**.

When the lincRNAs significantly more expressed in mixed-sex ovaries (bO) were analyzed, we found 64 lincRNAs in common in the pairwise comparisons with bT, bM, bF, and sO **(Fig P, red circle)** while looking at lincRNAs significantly more expressed in bT we found 141 in common in the pairwise comparisons with bO, bM, and bF and not with sT **(Fig Q, red circle)**, with only one additional lincRNA being more expressed in bT compared with sT and present in the other comparisons **(Fig Q, red circle)**.

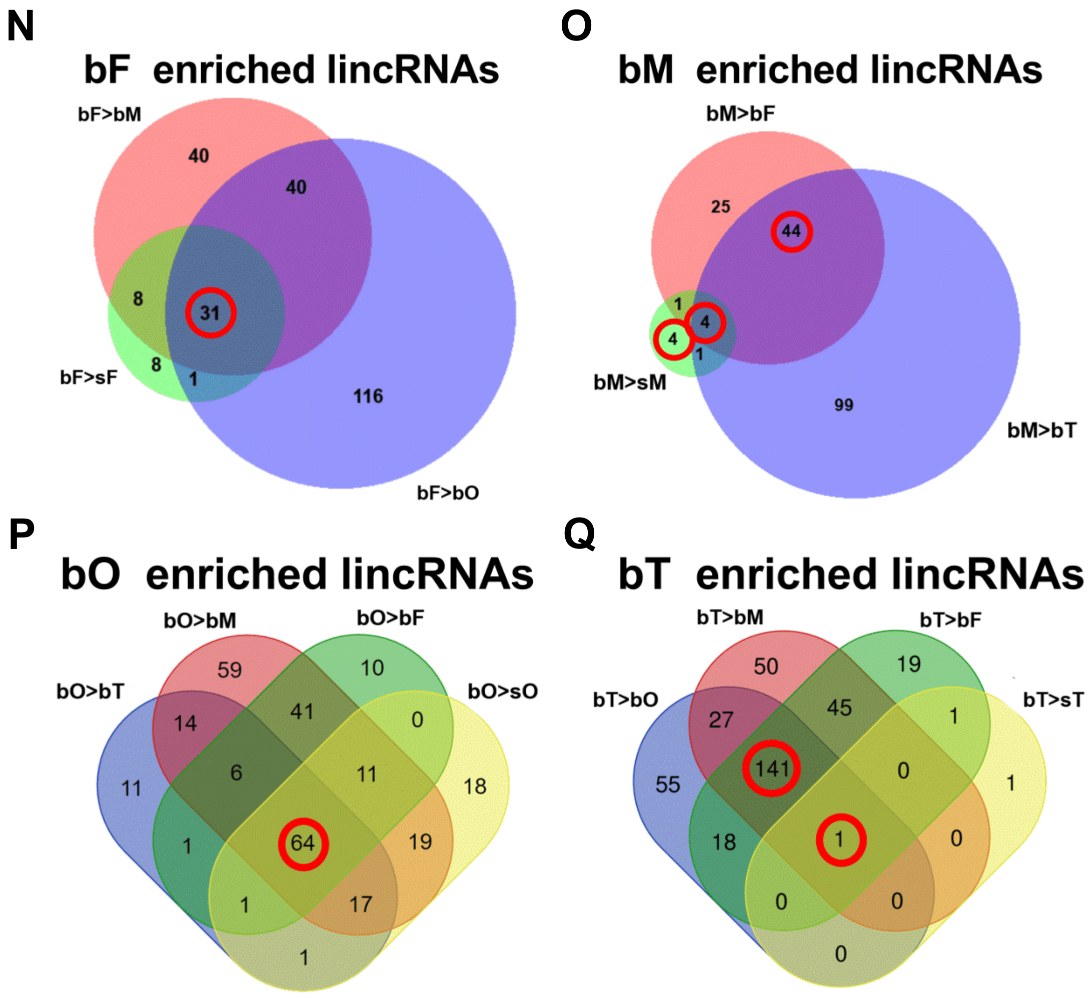

Figs N to Q. Analysis of gender and gonad enriched lincRNAs. The Venn diagrams show the number of lincRNAs that were detected as more expressed in: (**N**) mixed-sex females (bF) in pairwise comparisons with bM, sF, and bO and the circle highlights the number of lincRNAs detected in common in the three pairwise comparisons; (**O**) mixed-sex males (bM) in pairwise comparisons with bF, bT and sM, and the circles highlight the number of lincRNAs detected in common in the three pairwise comparisons, in one of the two pairwise comparisons, and in bM compared with sM; (**P**) ovaries from mixed-sex females (bO) in pairwise comparisons with bT, bM, bF and sO, and the circle highlights the number of lincRNAs detected in common in the four pairwise comparisons; (**Q**) testes from mixed-sex males (bT) in pairwise comparisons with bO, bM, bF and sT, and the circles highlight the number of lincRNAs detected in common in the first three pairwise comparisons, as well as in the four comparisons. The list of lincRNA ID numbers of all SmLINC RNAs highlighted in the red circles is given in **Table Y in S2 Appendix.**

We further extended the search to include all lincRNAs expressed at levels of TPM > 2 in mixed-sex females (bF) and in ovaries from mixed-sex females (bO). We separated the lincRNAs that had epigenetics marks at their TSS (**Fig R**) from those without the marks (**Fig S**) and identified the number of lincRNAs that were detected as significantly more expressed in bF in the pairwise comparisons with sF and bM or as

significantly more expressed in bO in the pairwise comparison with sO, as indicated in the Venn diagrams of **Figs R and S**.

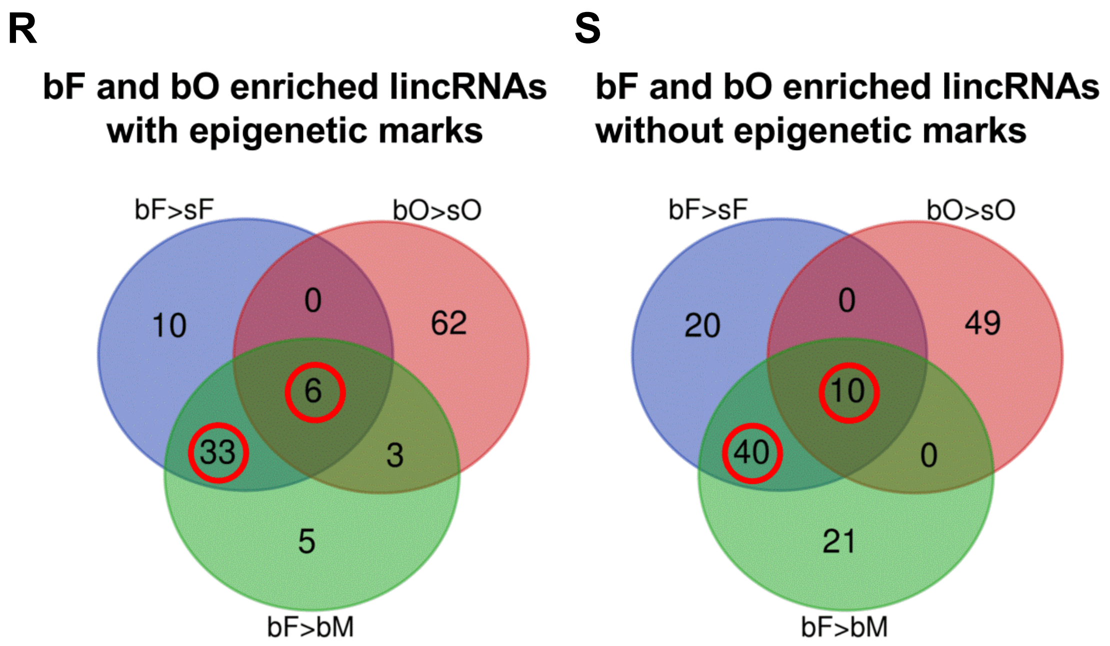

Figs R and S. Analysis of gender and gonad enriched lincRNAs. The Venn diagram shows the number of lincRNAs that were detected as more expressed in: (**R**) mixed-sex females (bF) in pairwise comparisons with bM and sF, and in ovaries from mixed-sex females (bO) compared with single-sex (sO), in lincRNAs with epigenetic marks at the TSS. (**S**) mixed-sex females (bF) in pairwise comparisons with bM and sF, and in ovaries from mixed-sex females (bO) compared with single-sex (sO), in lincRNAs without epigenetic marks at the TSS. The circles highlight the number of lincRNAs detected in common in the three pairwise comparisons and in one of the two pairwise comparisons. The list of lincRNA ID numbers of all SmLINC RNAs highlighted in the red circles is given in **Table Y in S2 Appendix.**

We narrowed the above selected sets by excluding the lincRNAs with more than one isoform in their genomic locus, resulting in a total of 33 lincRNA candidates containing a chromatin epigenetic mark at their transcription start site (TSS), an indicative of lincRNA regulation (**Table Z, upper panel**) and 28 lincRNA candidates without the epigenetic mark at their TSS (**Table Z, lower panel**).

Table Z. Number of lincRNAs selected for RT-qPCR validation and phenotypic assays validation[a].

Enriched Condition	Number of lincRNAs with epigenetic marks around TSS	Number of lincRNAs selected for further investigation	2<TPM>5	TPM>5
bF	10	4	1	3
bM	3	1		1
bO	7	1		1
bT	13	2	1	1
Total	33	8	2	6

Enriched Condition	Number of lincRNAs without epigenetic marks around TSS	Number of lincRNAs selected for further investigation	2<TPM>5	TPM>5
bF	10	2		2
bM	-	-		
bO	4	0		
bT	14	0		
Total	28	2		2

[a] LincRNAs identified as being more expressed in common in the pairwise comparisons highlighted in Figures B to G were further selected for RT-qPCR validation based on the level of expression above 2 transcripts per million (TPM) or above 5 TPM, and on the presence of only one isoform in their genomic locus. They were further divided into the ones having epigenetic marks at their Transcriptional Start Site (TSS) (**upper panel**) or not having the marks (**lower panel**), as annotated by Maciel et al. 2019 [5]. Finally, lincRNAs were selected for further investigation based on our ability to design specific sets of primer pairs for RT-qPCR that passed the 80% primer efficiency threshold. The list of lincRNA ID numbers of all SmLINC RNAs indicated in this table is given in **Table Y in S2 Appendix.**

Finally, we excluded from the list of candidates those for which no specific pairs of primers for PCR could be designed, or those for which the primer pairs did not pass a 80% primer efficiency threshold. The final set of lincRNAs selected for further investigation **(Table Z)** comprised 10 transcripts, being 7 bF enriched lincRNAs**,** 1 bM

enriched lincRNA, 1 bO enriched lincRNA, and 1 bT enriched lincRNA, which are described in detail in the main text.

3. Figs A to H and Legends

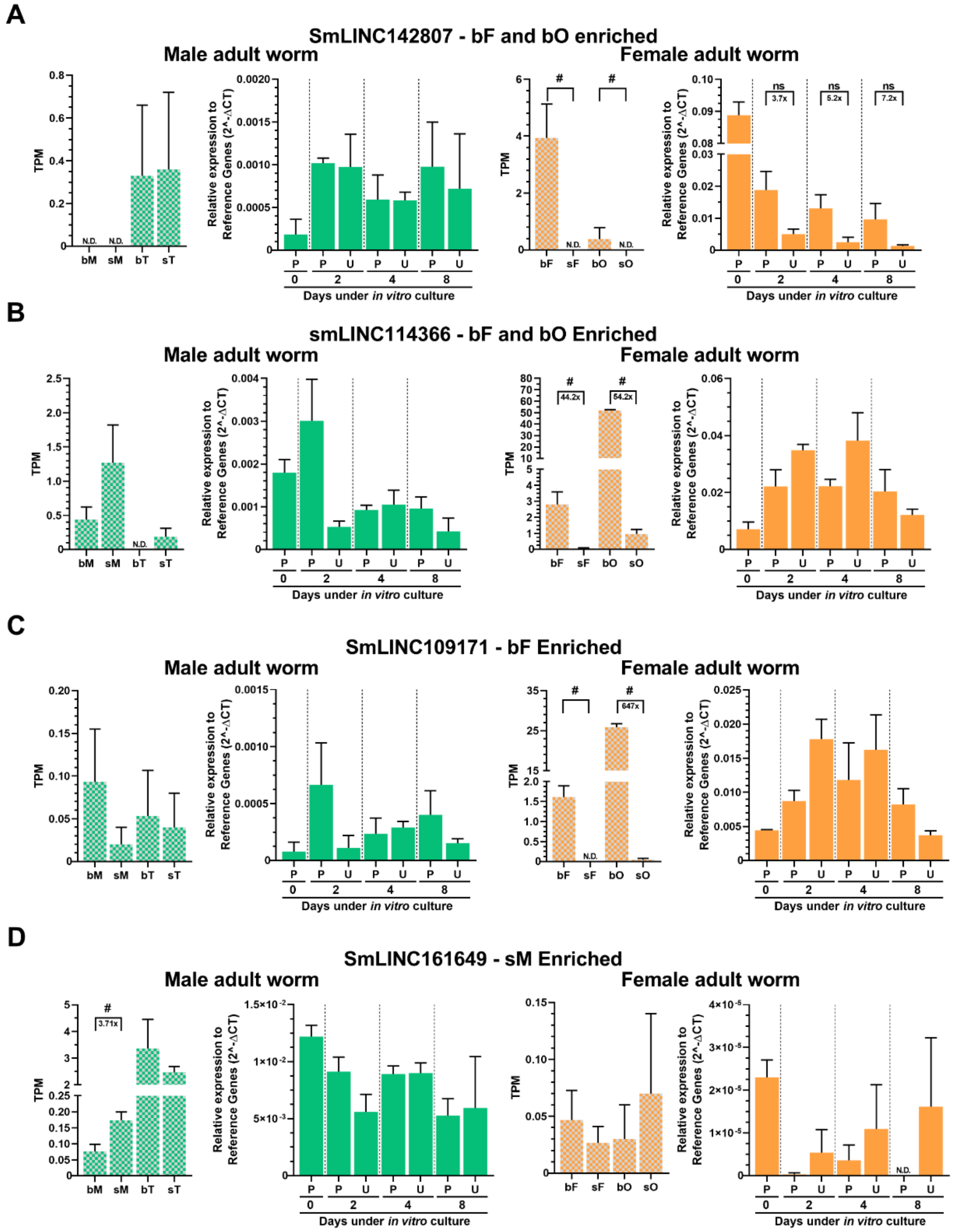

Fig A. Expression validation of lincRNAs enriched in four different male and female samples in the RNA-Seq analyses and measured by RT-qPCR in *S. mansoni* cultured *in* vitro for 2, 4 or 8 days as paired couples or unpaired males and females. Four lincRNAs detected as enriched in the re-analyses of the RNA-Seq dataset of Lu et al., 2016 [1], were selected for RT-qPCR assays, namely (**A**) SmLINC142807, enriched in

bF and bO; (**B**) SmLINC114366, enriched in bF and bO; (**C**) SmLINC109171, enriched in bF; and (**D**) SmLINC161649, enriched in sM. Male related results are shown on the left (green) and female results on the right (orange). Paired couples (P) or unpaired (U) parasites were obtained by perfusion of hamsters infected for 42 days with *S. mansoni* cercariae. After perfusion, males and females were cultured *in vitro* for 2, 4 or 8 days as paired (P) couples or unpaired (U) worms. RT-qPCR results (solid-colored graphs) are normalized to the geometric mean of reference genes Smp_099690 and Smp_023150. Expression values from 4 different biological replicates are shown. Standard error of the mean (SEM) is shown in the error bars. N.D.: Not detected. ns: p-value > 0.05, Student t test. For comparison, RNA-Seq data from re-analysis of Lu et al., 2016 [1] is shown (plaid-colored graphs) and the expression is measured in TPM (transcripts per million); RNA-Seq data is retrieved from males (M), females (F), testes (T) or ovaries (O) from either a mixed-sex (b) or a single-sex (s) infection; (#) = FDR<0.005. The fold-change differences between the compared groups are represented under the brackets.

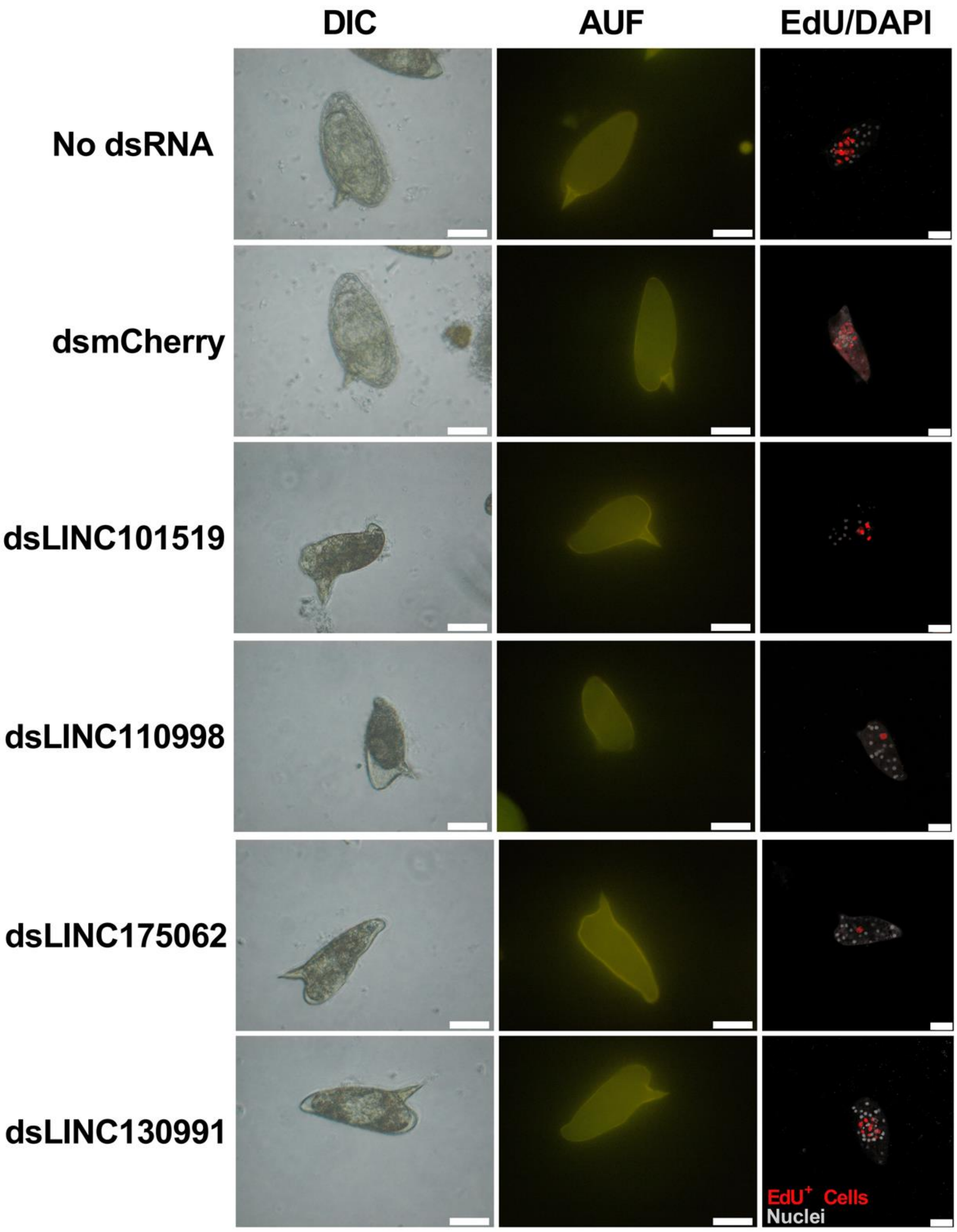

Fig B. *Schistosoma mansoni* egg phenotypes upon lincRNAs silencing. Adult worms retrieved from perfusion of 42 days-infected Syrian hamsters were cultivated in 6-well plates with 5 mL of ABC media for eight days without dsRNA, with dsRNA targeting mCherry (control dsRNA), or with dsRNA targeting SmLINC101519, SmLINC110998, SmLINC175062 or SmLINC130991, as indicated at left. Note that SmLINC130991 is an unrelated control lincRNA, not detected as enriched in mixed-sex samples in the mixed-sex/single-sex *in vivo* model comparison. Eight days after treatment, the eggs were counted and collected for images acquisition and further characterization. Egg area, autofluorescence (AUF), and proliferating cells (EdU^+)/total cell (DAPI, nuclei) number

ratio, were measured by using Fiji/ImageJ as described in Methods. For the EdU and DAPI labeling, EdU was added to the collected eggs and incubated in ABC medium for another day; DAPI was added 4h before image acquisition. After that, the eggs were processed and EdU (red) and DAPI (gray) fluorescence were measured as described in Methods. Representative images from 1 out of 4 experiments with n > 10 eggs. Note that for each dsRNA that is shown, a different egg in the field is shown at each of the different assays that are presented. Scale bars: 20 µm for egg area and autofluorescence (AUF) and 25 µm for $DAPI^+$ and $EdU^+/DAPI^+$ eggs.

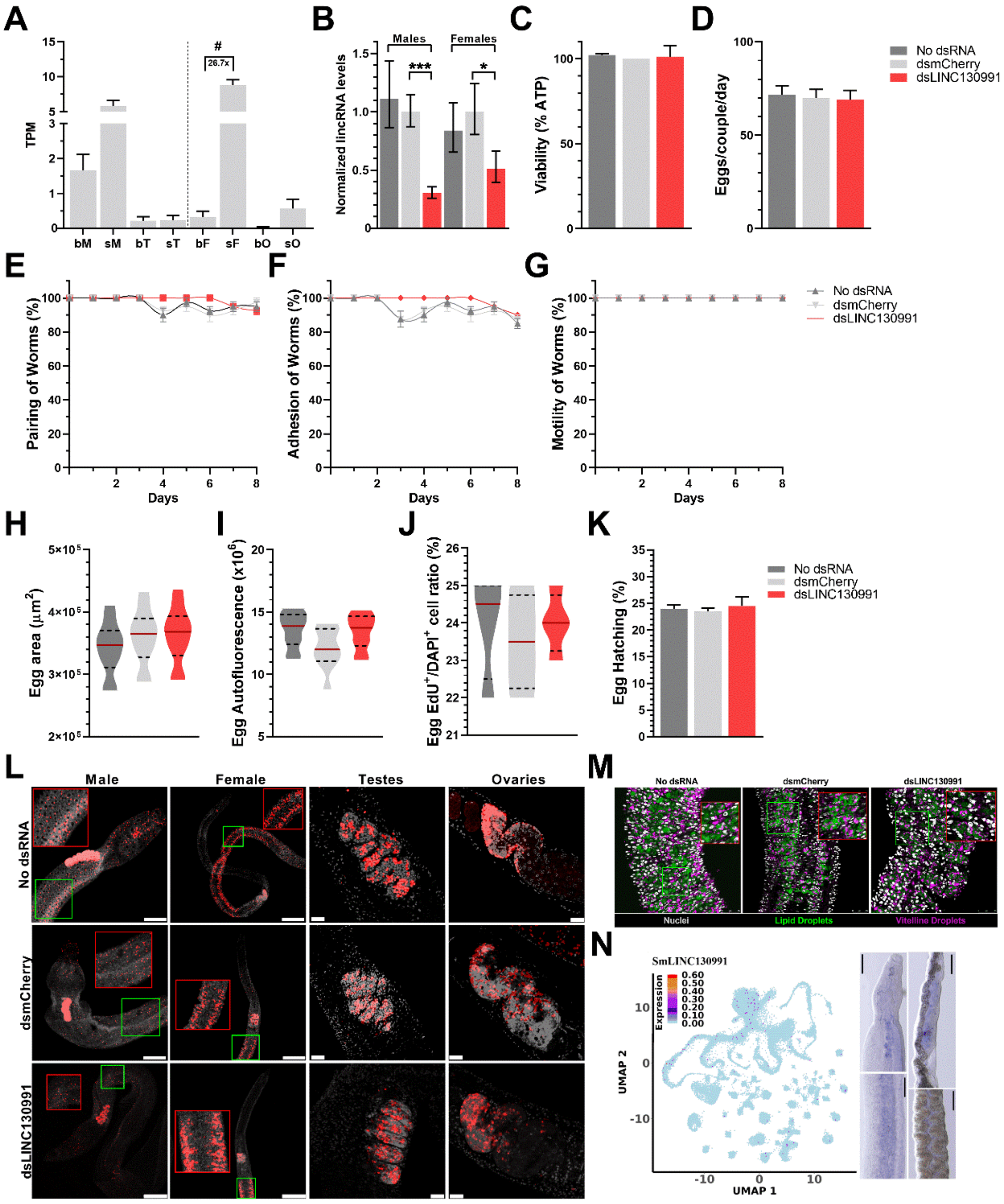

Fig C. Lack of phenotypic changes in *Schistosoma mansoni* adult worm couples upon *in vitro* silencing of SmLINC130991, an unrelated control lincRNA. Paired couples were obtained by perfusion of Syrian hamsters infected for 42 days with *S. mansoni* cercariae. Couples were cultured *in vitro* for 8 days, in ABC media supplemented with 30 µg/mL of dsRNA targeting SmLINC130991 (red bars in panels B to K), an unrelated control that was not enriched in any of the mixed-sex samples in comparison with the single-sex samples as shown below in panel (A). Medium was exchanged every other day while dsRNA was added every day. dsRNA targeting mCherry (a gene that is not present

in *S. mansoni*) was assayed in parallel as a negative control (light gray bars). Results for parasites cultured with no dsRNA are also shown (dark gray bars). **(A)** RNA-Seq expression data for SmLINC130991 from the re-analysis of Lu et al., 2016 [1] is shown and the expression is measured in TPM (transcripts per million); RNA-Seq data is retrieved from males (M), females (F), testes (T) or ovaries (O) from either a mixed-sex (b) or a single-sex (s) infection; (#) = FDR<0.005. The fold-change difference between the compared groups is represented under the bracket. Note that this lincRNA was chosen as an unrelated control because it was not enriched in any of the mixed-sex (b) samples. (**B**) RT-qPCR results for SmLINC130991 expression level are normalized to the geometric mean of reference genes Smp_099690 and Smp_023150. **(C)** Viability of adult worms (males+females) was monitored using the ATP-Glo Assay. **(D)** At the end of the experiment (8 days), eggs were collected and counted. **(E-G)** Pairing status, adhesion to the plate and motility of worm couples were traced along the 8 days of the experiment. **(H-K)** Collected eggs were monitored for their size (area) **(H)**, integrity of their eggshell (autofluorescence) **(I)**, proliferation status of the embryos (Egg EdU+/DAPI+ cell ratio) **(J),** and the percentage of egg hatching was measured by keeping the eggs in culture for another 7 days in ABC media for synchronization of their development, then assessing egg hatching as described in Methods, with the percentage of hatched eggs being shown **(K)**. Violin plot representation at figures **(H-J)** with the median indicated by the red line and the quartiles represented by the dashed lines. (**L**) EdU detection was performed as described in the Methods. DAPI stained cells nuclei are in gray and EdU+ cells (proliferating cells) are stained in red. Scale bars: 250 µm for the adult worm images (Males and Females), and 25 µm for the adult worm gonad images (Testes and Ovaries). (**M**) Female vitellaria stained with Fast Blue BB (pink) and BODIPY (green), which labeled vitelline and lipid droplets in the vitellaria, respectively. DAPI staining of cells nuclei is shown in gray. Scale bars: 25 µm. (**N**) WISH of SmLINC130991 is shown as the blue color stains in the male (left) or female (right) adult worm images of the heads and bodies. Scale bars are 100 µm. SmLINC130991 expression patterns across single-cell clusters are shown with a UMAP plot, which is colored by gene expression level (blue = low, red = high) and the scale represents log10(UMIs+1). The red borders in all microscopy images define zoomed-in insets of interest that correspond to the regions within green borders. Representative microscopy images from 3 experiments with n > 10 parasites. Quantitative data from 4 different biological replicates is shown. Standard error of the mean (SEM) is shown in the error bars in (**B**). (*) = $p < 0.05$; (***) = $p < 0.001$, Student t test.

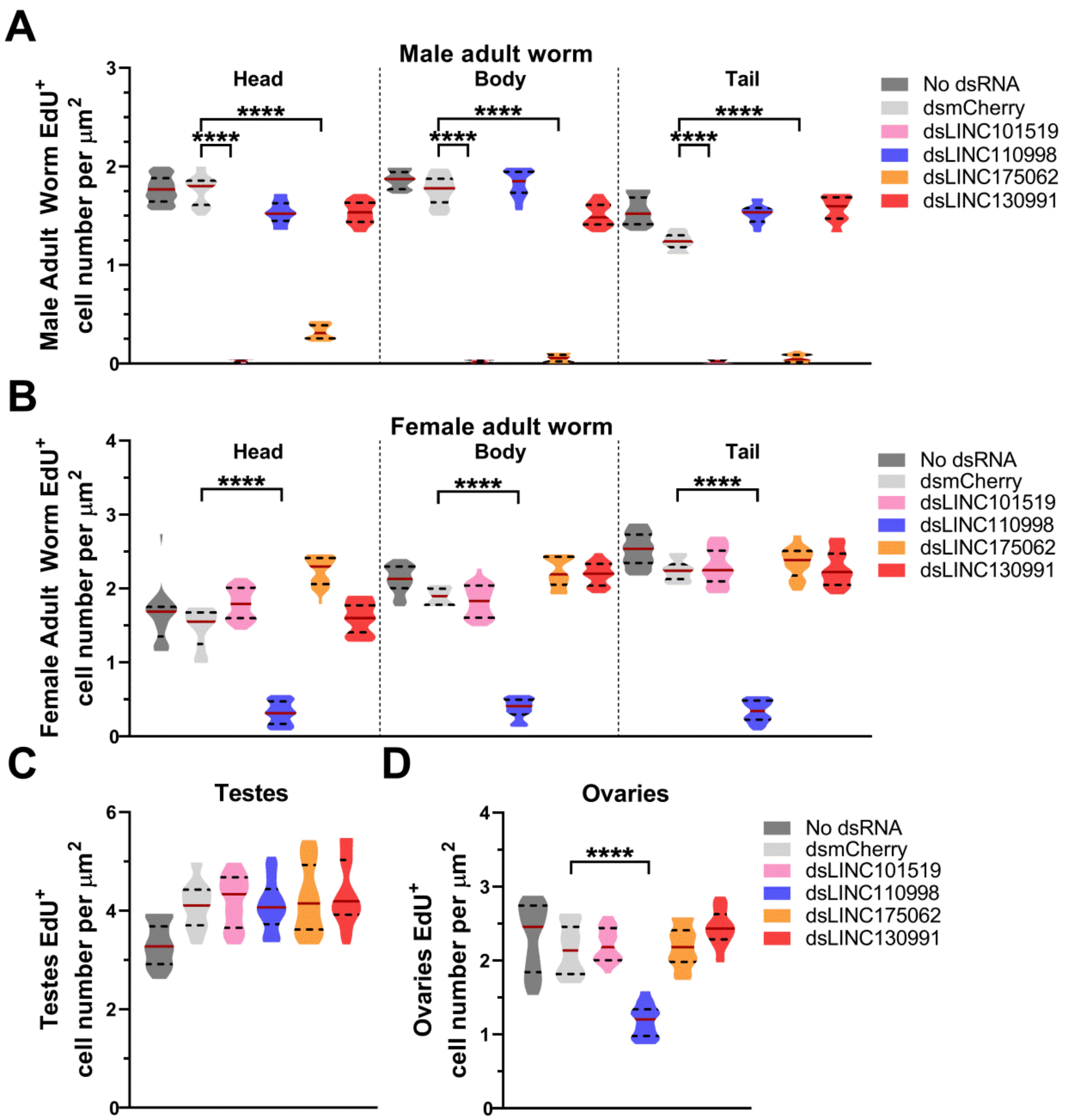

Fig D. *In vitro* silencing of pairing-dependent lincRNAs in *S. mansoni* adult worm couples leads to an impaired cell proliferation status. Paired adult worm couples were cultured *in vitro* for 8 days in ABC media and were pulse labelled with EdU that was added on the 7th day and cultured for an additional 24h, as described in Fig. 6. EdU fluorescence images were acquired and quantified with Fiji/Image J as described in Methods, and quantification is shown as the number of EdU^{+} cells per µm^{2} in panels A to D. Cultured media were supplemented with 30 µg/mL of dsRNA targeting each of the indicated lincRNAs, namely SmLINC101519, SmLINC110998, SmLINC175062, or SmLINC130991, with a negative control dsRNA targeting mCherry (a gene that is not present in *S. mansoni*), or with no dsRNA. Note that SmLINC130991 is an unrelated control lincRNA, not detected as enriched in mixed-sex samples in the mixed-sex/single-sex *in vivo* model comparison. EdU labeling quantification represented by the violin plots in (**A**) male adult worms, (**B**) female adult worms, and in (**C and D**) their gonads (Testes (**C**) and Ovaries (**D**)). Median number of EdU^{+} cells per µm^{2} indicated by the red line and the quartiles represented by the dashed lines. Results from 4 different biological replicates

are shown with n = 7 parasites for each replicate. (****) = $p < 0.0001$, One-Way Welch ANOVA test with Holm-Sidak's multiple comparisons to dsmCherry group was used.

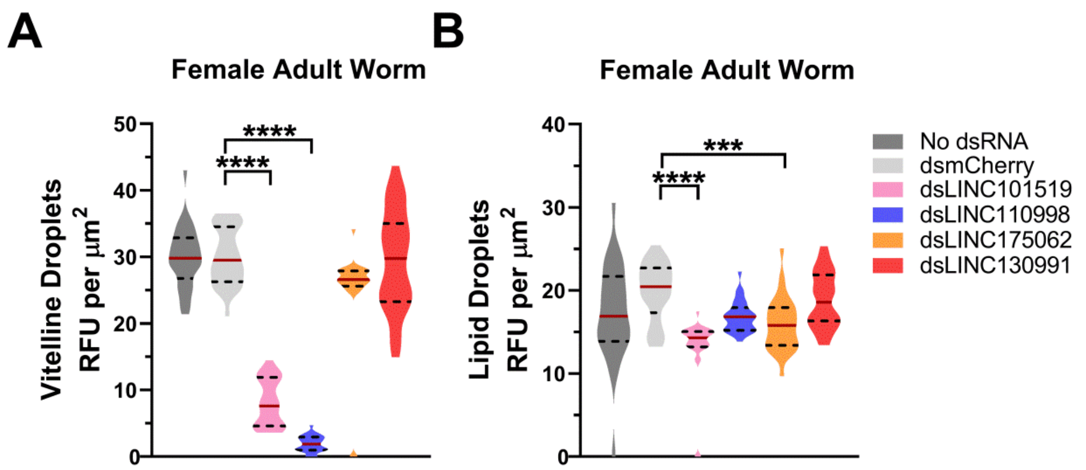

Fig E. *In vitro* silencing of pairing-dependent lincRNAs in *S. mansoni* adult worm couples causes female vitellaria impairment. Paired couples were obtained by perfusion of Syrian hamsters infected for 42 days with *S. mansoni* cercariae. Couples were cultured *in vitro* for 8 days in ABC media supplemented with 30 µg/mL of dsRNA targeting each of the indicated lincRNAs, namely SmLINC101519, SmLINC110998, SmLINC175062 or SmLINC130991, with a negative control dsRNA targeting mCherry (a gene that is not present in *S. mansoni*) or with no dsRNA. Note that SmLINC130991 is an unrelated control lincRNA, not detected as enriched in mixed-sex samples in the mixed-sex/single-sex *in vivo* model comparison. Medium was exchanged every other day while dsRNA was added every day. Female vitellaria were stained with Fast Blue BB and BODIPY as described in Methods, which labeled vitelline and lipid droplets in the vitellaria, respectively. Fluorescence images were acquired and quantified with Fiji/Image J as described in the Methods, and quantification is shown in panels **A** and **B** as Relative Fluorescence Units (RFU) per μm^2. Vitellaria quantification represented by the violin plots in (**A**) for the vitelline droplets and (**B**) for the lipid droplets. Median RFU per μm^2 indicated by the red line and the quartiles represented by the dashed lines. Results from 4 different biological replicates are shown with n = 7 parasites for each replicate. (***) = $p < 0.001$; (****) = $p < 0.0001$, One-Way Welch ANOVA test with Holm-Sidak's multiple comparisons to dsmCherry group was used.

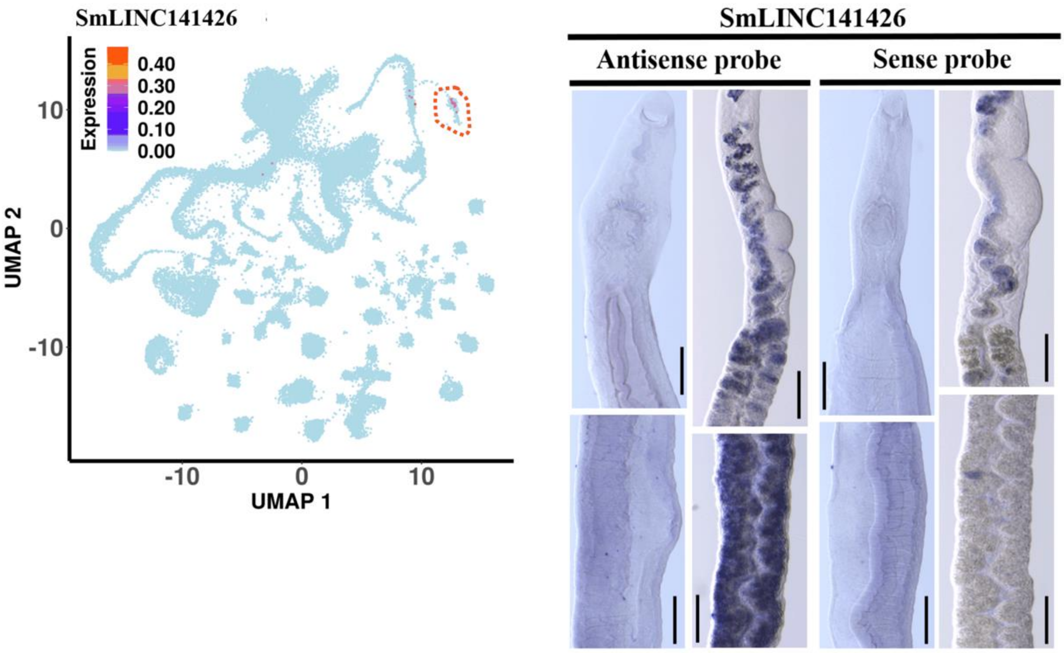

Fig F. Localization of SmLINC141426 in adult worm tissues by whole mount *in situ* hybridization. Whole mount *in situ* hybridization (WISH) of SmLINC141426 is detected with the antisense probe, as indicated by the legend at the top; adult male (left) or female (right) worm images of the heads and bodies are shown. Hybridization is shown as the blue color stains. Scale bars are 100 µm. Negative control WISH images obtained with the sense probe are shown at the right, as indicated by the legend at the top. For comparison purposes, single-cell RNA-Seq data from Morales-Vicente et al., 2022 [6] was retrieved (http://verjolab.usp.br:8081/). LincRNA expression pattern is shown with an UMAP plot, which is colored by gene expression (blue = low, red = high) and the scale represents log10(UMIs+1). Region enclosed by the red dashed line indicates the male gametes cell cluster, where a few cells expressed the lincRNA.

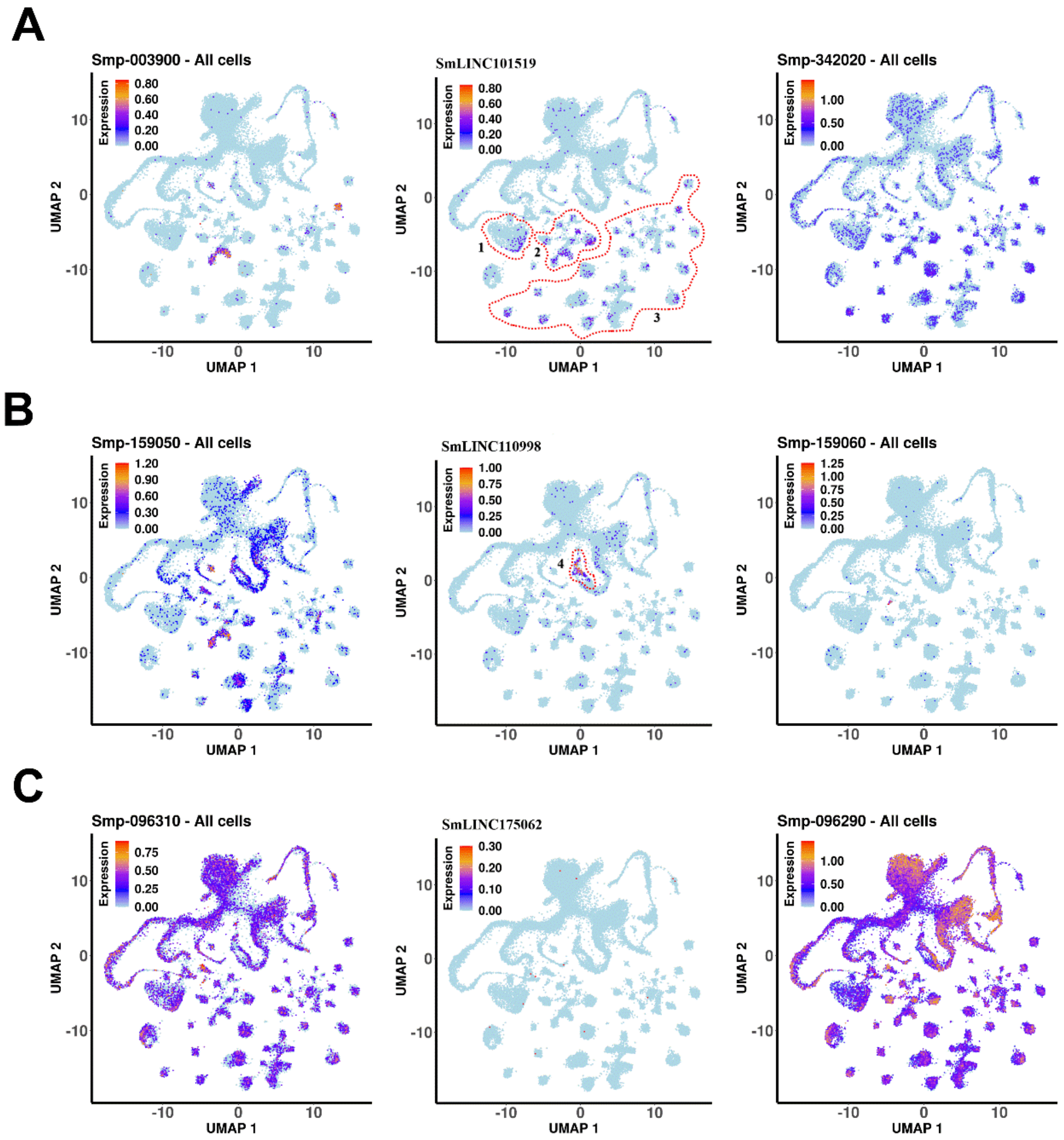

Fig G. UMAP plots of expression levels of lincRNAs involved in pairing status, with their corresponding *cis*-neighboring protein-coding genes (up- and downstream in the lincRNA genomic locus). Single-cell RNA-Seq data was retrieved from Morales-Vicente et al., 2022 [6] (see http://verjolab.usp.br:8081/). The lincRNA and Smp expression patterns are represented by UMAP plots. On the left panel, the upstream neighbor protein-coding gene is shown; the lincRNA is shown in middle panel; and on the right, the downstream neighbor protein-coding gene is shown. **(A)** SmLINC101519, and the red dotted lines mark Region 1, parenchyma 1 cells cluster; Region 2, muscle cell clusters; Region 3, neuron cell clusters; **(B)** SmLINC110998, and the red dotted line indicates Region 4, late vitellocyte cells cluster; and **(C)** SmLINC175062.

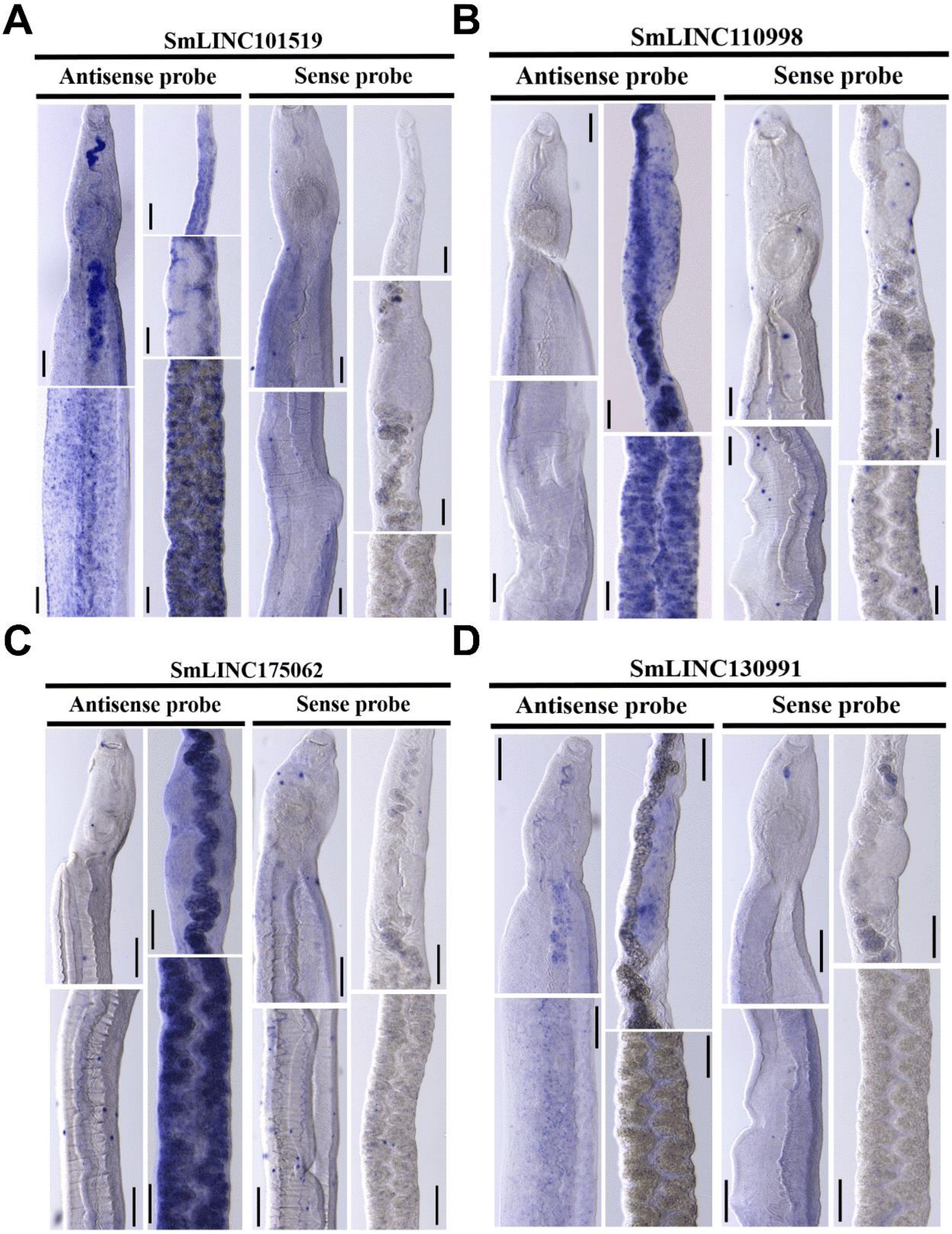

Fig H. Negative control of whole mount *in situ* hybridization with sense probes for four selected lincRNAs. Whole mount *in situ* hybridization (WISH) for each of 4 lincRNAs was assayed in adult worm tissues with both the antisense probe (positive control) and the sense probe (negative control), as indicated at the top of each image. (**A**) SmLINC101519, (**B**) SmLINC110998, (**C**) SmLINC175062, and (**D**) SmLINC130991. Note that SmLINC130991 is an unrelated control lincRNA, not detected as enriched in mixed-sex samples in the mixed-sex/single-sex *in vivo* model comparison. Hybridization is shown as the blue color stains in the male (left) or female (right) adult worm images of the heads and bodies. Scale bars are 100 µm.

References

1. Lu Z, Sessler F, Holroyd N, Hahnel S, Quack T, et al. (2016) Schistosome sex matters: a deep view into gonad-specific and pairing-dependent transcriptomes reveals a complex gender interplay. Sci Rep 6: 31150.
2. Haeberlein S, Angrisano A, Quack T, Lu Z, Kellershohn J, et al. (2019) Identification of a new panel of reference genes to study pairing-dependent gene expression in *Schistosoma mansoni*. Int J Parasitol 49: 615-624.
3. Vandesompele J, De Preter K, Pattyn F, Poppe B, Van Roy N, et al. (2002) Accurate normalization of real-time quantitative RT-PCR data by geometric averaging of multiple internal control genes. Genome Biol 3: RESEARCH0034.
4. Andersen CL, Jensen JL, Orntoft TF (2004) Normalization of real-time quantitative reverse transcription-PCR data: a model-based variance estimation approach to identify genes suited for normalization, applied to bladder and colon cancer data sets. Cancer Res 64: 5245-5250.
5. Maciel LF, Morales-Vicente DA, Silveira GO, Ribeiro RO, Olberg GGO, et al. (2019) Weighted gene co-expression analyses point to long non-coding RNA hub genes at different *Schistosoma mansoni* life-cycle stages. Front Genet 10: 823.
6. Morales-Vicente DA, Zhao L, Silveira GO, Tahira AC, Amaral MS, et al. (2022) Single-cell RNA-seq analyses show that long non-coding RNAs are conspicuously expressed in *Schistosoma mansoni* gamete and tegument progenitor cell populations. Front Genet 13: 924877.
