# Supplementary material for: Long non-coding RNAs are essential for Schistosoma mansoni pairing-dependent adult worm homeostasis and fertility
Source: PLoS Pathog. 2023 May 5;19(5):e1011369. doi: 10.1371/journal.ppat.1011369 (PMC10191282; doi:10.1371/journal.ppat.1011369)
Supplement: Attachment — Submitted filename: Authors responses to the reviewers reports-23mar2023.docx [file ppat.1011369.s004.docx]

**Responses from the authors to the Reviewers’ queries**

**Part I - Summary**
Please use this section to discuss strengths/weaknesses of study, novelty/significance, general execution and scholarship.

***Reviewer #1:*** *The authors tried to understand the roles of long non-coding RNAs in male and female pairing for Schistosoma mansoni. Certainly, the manuscript focuses on a potentially interestingly topic for Schistosoma. Unfortunately, the manuscript suffers from several weaknesses.*

*1. Line 128: As indicated,16583 lncRNAs were identified, however, it was not clear that why the authors choose the 12 lincRNAs for further analysis. Since lincRNAs usually display tissue- and temporal-specific expressions, the authors utilized the RNA-seq data published by Lu et al , in which the adult worms collected from 42-67 days of post infection were used. Although the authors tried to validate the expression of 12 selected lncRNAs, it is unclear which stage of parasites were used. Obviously, the RT-qPCR analysis of expression of lncRNAs were lack of systematic validation currently.*

In fact, we indicated in the second paragraph of the Results that 16,583 lncRNAs were identified as expressed in mixed-sex/single-sex parasites according to our reanalysis of Lu et al. 2016 data (lines 134-146). In this regard, we would like to mention that we also indicated in the third paragraph that, by comparing the expression of mixed-sex and single-sex parasites, we identified 3681 unique differentially expressed (DE) lncRNA genes, which were shown in Fig. 2A (lines 147-155). And immediately after that, we had extensively described under the topic “**Selection of lincRNAs to be tested for their involvement in adult worm pairing”** (lines 160ff) the criteria for selection of the 10 lincRNA candidates among the DE lncRNA genes, along with a detailed description of the comparative steps performed for the selection, which were given in the **Supplementary Methods in S1 Text**. The criteria for selection of the lincRNA candidates among the DE lncRNA genes was already present in the original version of the manuscript; however, we have re-written some sentences in order to make them clearer (lines 160 – 175).

Validation of Lu et al. RNA-seq expression data for all 10 selected lincRNAs was assayed by us with RT-qPCR using the same adult worms’ stage as in the original paper, as shown in Figs. 3, 4, and Supplementary Fig. 1. In all these figures it had already been clearly indicated on top of each panel that “Male adult worm” or “Female adult worm” were assayed, and the results of RT-qPCR were given for each lincRNA as measured for paired (P) or unpaired (U) parasites at 0, 2, 4 and 8 days in culture.

We have re-phrased some of the sentences in the first three topics of the Results, namely “**Identification of lncRNAs differentially expressed between immature and mature *S. mansoni* parasites and their gonads”** (lines 117-155)**, “Selection of lincRNAs to be tested for their involvement in adult worm pairing”** (lines 160-175), and “**Validation by RT-qPCR of differential expression of the selected lincRNAs upon worms unpairing”** (lines 176-197) to make sure that their contents are better conveyed to the readers.

2. *The authors used dsRNA to silence the lncRNAs and then evaluate the effect by using Edu staining, the authors claimed that cell proliferation and vitellaria composition were potentially regulated by the lncRNAs. Obviously, the Edu staining in different treatments was lack of normalization. For example, it is possible that the authors use DAPI to stain cell to normalize the Edu staining. If so, the conclusion should be stronger.*

As requested by the Reviewer, we have now quantified cell proliferation in male and female adult worms head, body, and tail, as well as in testes and ovaries, as measured by the number of EdU^+^ cells per μm^2^ (new Supplementary Figure 4 in S1 Text). We also quantified the extent of vitelline droplets and lipid droplets in the vitellaria, as measured by Relative Fluorescence Units per μm^2^ (new Supplementary Figure 5 in S1 Text). This is described on lines 306-310, 314-320, 321-326, and 327-341, 345-348.

3. *Line 292-297, there were several contradictory statements/observations between Wish and single cell sequence results. For examples, the authors mentioned that SmLINC101519 was expressed in the female uterus, vitellaria and around the female ovary, However, based on the Wish results, SmLINC101519 was not significantly enriched in female ovaries, the results contradicted the statements. In addition, the authors should figure out the possible reasons causing the inconsistent results between WISH and single cell sequence and then selected one confidence method to draw the related conclusions.*

We thank the Reviewer for pointing to the apparent inconsistency between WISH and single-cell RNA-Seq (scRNA-Seq) results for SmLINC101519 (which are now on lines 357-365). There are several possible reasons for the different results regarding localization of an RNA obtained with these two different methods. One of the main sources of difference is that scRNA-Seq depends on the efficient dissociation of intact cells across the different parasite tissues and on the efficient tagging and recovery of RNA from each isolated cell; in fact, only a median of 896 to 1487 genes per cell (including protein-coding and lncRNA genes) were detected as expressed across the different scRNA-seq samples in the work of Morales-Vicente et al. 2022, and it is conceivable that not all cell types and tissues were recovered at the same extent. Each of the two methods has pros and cons, and a comparison of the two may point to different clues regarding localization and possible function, but we do not think that we can point to one of them as better than the other. In fact, lack of validation of scRNA-Seq has been previously reported for specific markers of some cell clusters in *S. mansoni* (Diaz Soria et al.,2020.https://doi.org/10.1038/s41467-020-20092-5), and it has been speculated that signals from damaged or dying cells caused by laboratory procedures may be involved, since they are challenging to eliminate.

Regarding the SmLINC101519 findings, we decided to remove from our original text the expression “curiously, this [WISH localization] was not completely consistent” with the results from the single-cell RNA-Seq (scRNA-Seq) analysis, in which SmLINC101519 was detected in parenchyma, muscle and neuron cell clusters. Now, we only describe the scRNA-Seq detection in parenchyma, muscle, and neuron cell clusters. In addition, we add a possible explanation which is that this lincRNA might not be expressed in oocytes but rather in the muscle cells surrounding the ovary as well as in the muscle layer of the uterus. Please see lines 357-365.

4. *More importantly, the manuscript is lack of mechanism study of lncRNAs for regulating schistosome pairing and egg production.*

We would like to argue that publishing the information provided in our present work will pave the way for our group as well as for several other groups in the field to explore the possible molecular mechanisms of action of the different lincRNAs in *Schistosoma mansoni*.

5. *The manuscript also suffer from poor organization, unclear descriptions and others*.

Following this comment of Reviewer #1, as well as the specific suggestions for reorganization given on Part III below by Reviewer #2, we have now re-organized the section “**Validation by RT-qPCR of differential expression of the selected lincRNAs upon worms unpairing”**. We have now moved part of this section to Results (lines 178 to 197) and another part to the Supplementary Data in S1 Text (lines 23-85 in S1 Text). Essentially, we have placed a short version of testing the unpairing model with protein-coding genes under Results (lines 185-192 in the main text), and the full version of the results with protein-coding genes as Supplementary Data in S1 Text (lines 23-85 in S1 Text). We kept a short description of the procedures involved with the “**Parasite *in vitro* mimetic model of paired and unpaired adult worms”** in the Methods section (lines 638-653), while the results of protein-coding genes expression that were obtained as a control for characterization of the model and were originally in the Methods, have now been moved to the Results section (lines 185-192 in the main text) and to Supplementary Data in S1 Text (lines 23-85 in S1 Text), as described above.

***Reviewer #2:*** *The manuscript by Silveira et al. extends our knowledge on long non-coding RNAs of Schistosoma mansoni. After previous publications of the group on the expression of lncRNAs across the life-stages and across single-cells, now a re-analysis of publicly available RNAseq data of different sexes and gonads is presented. Among the lncRNAs, the focus was then set on the group of long intergenic non-coding (linc) RNAs. An impressive amount of data is presented that covers the functional characterization of several lincRNAs using basically all methods commonly used in schistosome research (RNAi, WISH, single-cell data analysis, EdU and other cell assays). The obtained results suggest a role of the selected lincRNAs in pairing stability, cell proliferation, vitellarium homeostasis and egg development. Finally, in vivo experiments using RNAi in infected mice were conducted which revealed a reduced number of worm pairs.

The study is well conducted and well written, and I am personally impressed by the amount of data. Apart from some requests for small modifications (see below), I like to raise one concern regarding the “in vitro mimetic model“ and one regarding the interpretation of pairing-dependent phenotypes:

1.) An in vitro mimetic model was used to validate the RNAseq data (Lu et al) on differential expression of selected genes between parasite sexes and pairing states in own qPCR analyses. Instead of the analysis of single-sex worms, this model involved the culture of worms, which were found separated upon perfusion, for a couple of days, in order to led them de-differentiate. This might be seen critical at first sight: it is unclear for how many days those worms were already separated in the hamster, or whether they freshly separated during perfusion. This way, worms with a different differentiation level enter the same experimental group. However, as long as the variance of gene expression between replicates is reasonable (which seems to be the case if I see Fig. 3 and 4), I would agree that this model is useful to learn about pairing-dependent processes.*

We thank the Reviewer for these insightful comments about the *in vitro* mimetic model that we used. In this regard, we would like to point that among the naturally unpaired worms that were recovered in the perfusion, a fraction of approximately 5–10% were smaller female worms with less hemozoin pigment, and they were discarded; only worms that exhibited a mature phenotype upon visual inspection were used for *in vitro* unpairing assays. We have now added this information to the Methods (lines 647-651). We have also included a few sentences in the Discussion about the uncertainty of the time when the parasites had separated, and the consideration that apparently this uncertainty did not have an impact on the assays (lines 440-447).

*2.) Seeing the high pair separation at the end of the experiment (70-90% separated, Fig. 5B), any conclusion on the effect of RNAi on the differentiation status of females (vitelline and lipid droplet staining; EdU+ cells) and their egg production is difficult. Of course, separated females will produce fewer eggs, cell proliferation will decline etc. I think one cannot be sure if the affected differentiation/egg production is just a consequence of the physical separation of females from their male partner, or if it is indeed pointing to a role of the lincRNA in the female. Can the authors defend their conclusion that a role for reproduction holds true?
If those parameters would be affected in still paired females, the interpretation would be easier. Did the authors compare the vitelline/lipid/EdU stainings of the few females that were still paired at the end of the experiment with those that were separated? Were effects similar? It should be specified in the Methods section or the figure legends of Fig. 6 and 7 whether only separated females were assessed by fluorescence microscopy, or both, paired and separated ones.*

We have not compared the separated females with the paired females when analyzing EdU staining and vitelline/lipid droplets staining at day 8 after lincRNAs silencing. The fraction of 10-30% worms that remained paired was analyzed together with the unpaired ones in the assays of Fig. 6 and 7. In fact, after EdU labelling (7^th^ and 8^th^ days) the parasites that eventually remained paired were separated by a 2 min incubation with anesthetic Tricaine (the first step in the standard procedure for the assay), followed by processing for fluorescence images acquisition. This information has now been added to the Methods, as requested by the Reviewer (lines 809-818).

We recognize that there is a complex interplay between the different phenotypic parameters that were evaluated here, in determining the trajectory of sustained fertility and reproduction in paired worms. It is apparent that silencing each of three different lincRNAs in paired couples kept in culture for 8 days has affected worms’ homeostasis to different extents and with different time courses. Worms’ adhesion and motility started falling after 4 days of silencing (Figs. 5C, 5D), which may have impacted on the ability of couples to remain paired. Pairing has fallen to 50% after 7 days of silencing and on the 8^th^ day only 10 to 30% couple pairs remained among the three different sets of dsLincRNA-silenced worms (Fig. 5B). While physical separation of females from their male partners will play a role in reducing egg production and cell proliferation, our data suggest that reduced pairing is not the single drive for altered reproduction and that different lincRNAs silencing have altered reproduction homeostasis in different ways. Thus, silencing of SmLINC175062 caused 85% couple pairing reduction at the 8^th^ day (Fig. 5B) with a significant, however the smallest reduction in egg production of 25% (Fig. 5E) and no reduction in vitelline droplets (Supplementary Figure 5A in S1Text), while the eggs deposited by SmLINC175062-silenced females had the highest reduction in hatching (35%) (Fig. 5J). In contrast, silencing of SmLINC101519 caused 75% couple pairing reduction (Fig. 5B) with the highest reduction in egg production of 60% (Fig. 5E) and a significant reduction of 75% in vitelline droplets (Supplementary Figure 5A in S1Text), while the eggs deposited by SmLINC175062-silenced females had no reduction in hatching ability (Fig. 5J). Further identification of molecular mechanisms of action of these lincRNAs is warranted to elucidate the pathways affected in the worms and the dynamic changes in the observed, diverse phenotypes. We have now added this text to the Discussion on lines 482-503.

***Reviewer #3:*** *The paper identified several lncRNAs associated with pairing of Schistosoma mansoni and the authors used in culture and in vivo knock down approached to try and shed light on the possible functions of some of those lncRNAs. The data points towards involvement of these lncRNAs in several important biological processes such viability, motility, cellular proliferation in body and gonads the development of the vitellaria. Some of the lncRNAs studies could be linked with specific phenotypes, however, to the opinion of this reviewer some of the conclusions are overstated and are not directly supported by the data.*

Following the criticisms of Reviewer#3 we have now re-phrased some of the conclusions. Please see below our responses to the specific criticisms raised by Reviewer#3 in the next section, Part II, along with the changes that we have made.

**Part II – Major Issues: Key Experiments Required for Acceptance**
Please use this section to detail the key new experiments or modifications of existing experiments that should be absolutely required to validate study conclusions.

Generally, there should be no more than 3 such required experiments or major modifications for a "Major Revision" recommendation. If more than 3 experiments are necessary to validate the study conclusions, then you are encouraged to recommend "Reject".

**Reviewer #1:** (No Response)

**Reviewer #2:** None

**Reviewer #3:** Results:

*Fig - 5: It appears that the knock down of both DSLinc175062 and DSLinc110998 did not reduce viability while there is a significant reduction in motility. Thus, could it be that the reduction in adhesion and pairing is simply because of reduction in motility? how can we conclude that the it is a specific effect on coupling?*

We thank the Reviewer for raising this question. Indeed, silencing of SmLINC110998 and SmLINC175062 did not affect viability (Fig. 5E) while affecting motility (Fig. 5D). Therefore, we cannot rule out the possibility that aberrant motility also plays a role in reducing worm pairing. We have now added this interpretation to the Discussion (lines 467-471).

*It is also surprising to see that even though DSLinc124324 significantly reduced the viability of the worms, their egg production was not significantly changed? Can the authors explain this?*

As shown in the original Fig. 5F the egg production was significantly reduced by 45% upon SmLINC124324 silencing. Nevertheless, we have now excluded SmLINC124324 from the manuscript, because upon re-mapping the SmLINC124324-directed dsRNA probe against the new version 9 of the parasite transcriptome, some off-target matches were detected, as explained further below, and we could not ensure that we were targeting SmLINC124324 exclusively.

*Along this line…is it surprising that DSLinc124324 reduced body cell proliferation (Fig 6)? This should be expected when viability is reduced.*

Indeed, SmLINC124324 silencing had reduced female body cell proliferation as seen in the original Fig.6, along with a 60% reduction in viability (original Fig. 5E). As explained above, we have now removed SmLINC124324 data from the manuscript. As indicated by the Reviewer, reduction in viability along with reduction in body cell proliferation was not surprising as body cell proliferation might require a high supply of ATP and therefore was impacted when knockdown of SmLINC124324 caused the 60% reduction of available ATP in the parasite’s cells (original Fig. 5E).

In contrast, knockdown of SmLINC101519 caused a 40% reduction in viability (Fig. 5E) while showing a reduced egg cells proliferation (Fig. 5I) and a sustained female somatic stem cells proliferation (Supplementary Figure 4 in S1 Text, where the images are now quantified). This suggests that, when parasite homeostasis was affected by knockdown of SmLINC101519, egg and body cell proliferation might be the sources of highest ATP consumption, as opposed to worm motility. In fact, knockdown of SmLINC101519 caused the least effect on motility (Fig. 5D). On the other hand, motility was reduced by 60 to 90% upon knockdown of the other two lincRNAs (SmLINC110098 and SmLINC175062), which was not accompanied by reduction in viability (Fig. 5E), again suggesting that the effects on motility were not directly correlated with ATP levels. In a new paragraph that was added to the Discussion, we bring this point out (lines 467-481).

*In addition, it is not clear how the data in Fig 6 was quantified? i.e. the images presented are representation of how many worms? How did the EDU+ cells were quantified and compared with the controls?*

We have now included new Supplementary Figure 4 in S1 Text where the quantification of images in Fig. 6 is shown. We added a section to the Methods where we describe in detail the quantification with ImageJ and we indicate that a total of 28 images from each condition was quantified, from 4 different biological replicates (7 images per biological replicate). Lines 829-841.

*Given the differences in body cell proliferation between the sexes I am wondering if the reduced viability observed for DSLinc124324 and DSLinc101519 was measured for each of the sexes?*

In all cases, we have only measured viability for both sexes together.

*Also it appears that female body cells continue to proliferate in DSLinc101519 treatment even though the viability of this knock/down was reduced. Could it be that the reduced viability shown in Fig 5 is primarily attributed to males and that females were less affected? Sex dependent viability phenotype could contribute to the conclusions of this paper.*

We thank the Reviewer for calling the attention to a sex dependent differential effect of lincRNAs silencing on proliferative phenotype parameters. SmLINC101519 was the only lincRNA selected for silencing that was expressed at levels > 2 TPM in both males and females, enriched in mixed-sex males (bM) compared with single-sex males (sM), and the opposite, enriched in single-sex females (sF) compared with mixed-sex females (bF) (Fig. 4C). We measured body cell proliferation separately in each sex, and there was a clear sex difference in this phenotype. A marked decrease was observed in male body cell proliferation in dsLINC101519 treated worms (Supplementary Figure 4A in S1 Text), while no decrease was seen in females (Supplementary Figure 4B in S1 Text). Regarding viability, we believe that the reduced viability shown in Fig. 5E might primarily be due to consumption of ATP by the sustained egg and somatic cell proliferation in females when a loss of homeostasis occurs upon dsLINC101519 knockdown. Silencing of SmLINC175062 caused a similar decrease in male body cells proliferation (Supplementary Figure 4A in S1 Text), and no change in females. On the contrary, silencing of SmLINC110998 caused no change in males body cell proliferation (Supplementary Figure 4A in S1 Text), while leading in females to a marked decrease in body cell proliferation (Supplementary Figure 4B in S1 Text) and a decrease in vitelline droplets (Supplementary Figure 5A in S1 Text). It is apparent that these lincRNAs interfere in different ways, either directly or indirectly with proliferative and/or reproductive phenotypes, and identification of the molecular mechanisms that are involved should be pursued. We have now added a paragraph to the Discussion (lines 504-521).

*Fig. 7 – very nice data regarding DSLinc110998, but again since DSLinc124324 reduced viability what can we learn from the reduced vitelaria staining? Some kind of quantitative analysis is necessary*.

As requested by the Reviewer, we have now added a quantitative analysis of vitellaria staining; the procedure is described in the Methods (lines 842-852). We have now discussed the difference in reduced vitelline droplets upon silencing of certain lincRNAs together with the sex dependent reduction in body cell proliferation in the new paragraph added to the Discussion that is mentioned above (lines 504-521).

*Line 281 – “Taken together, these results show that lincRNAs are important for maintenance of adult worm pairing, viability, and fertility in vitro” this sentence is not necessarily accurate…some are important for viability other have impact on fertility and since those that do not affect viability affect motility maybe the aberrant motility is what reduces pairing?*

We thank the Reviewer for raising this important point. This sentence has been modified and moved from Results to the Discussion. In addition, the possibility that aberrant motility also plays a role in reducing worm pairing has now been included (lines 467-481).

*Fig 8 – The WISH results indicated that LINC175062 is expressed in mature vitellocytes. However, Fig 6 showed that its k/d had no or minor phenotype on the vitteline droplets, this is interesting and worth discussing when speculating about the role of these lncRNAs.*

We thank the Reviewer for pointing to this interesting comparison. According to the new quantification now shown in Supplementary Figure 5 in S1 Text, upon SmLINC175062 knockdown there was indeed no decrease in vitelline droplets (Supplementary Figure 5A) while a significant decrease in lipid droplets was observed (Supplementary Figure 5B), suggesting a possible role of this lincRNA on the vitellaria lipid metabolism. A sentence was added to the Discussion on lines 574-577.

**Part III – Minor Issues: Editorial and Data Presentation Modifications**
Please use this section for editorial suggestions as well as relatively minor modifications of existing data that would enhance clarity.

**Reviewer #1:** (No Response)

***Reviewer #2:*** *Line 69: The continously repeated statement that schistosomes are the only dioecious trematodes should no longer be used. Newer data identified a trematode species in crocodiles (Griphobilharzia amoena) that has a male and female individual (doi: 10.1645/GE-12-149.1). It is considered to belong to the Schistosomatoidea superfamily and to be a sister to the families of Spirorchidae and Schistosomatidae. Please rephrase this sentence (e.g. to „are the only mammalian trematodes“ or “are among the few trematodes“).*

Following the Reviewer recommendation, we changed the text on lines 70-72.

*Line 25 and lines 99f: The authors emphasize that a role of lncRNAs in maintaining the pairing status and female fertility is not yet known. It don’t think this is true. In their own paper (Silveira et al. 2002, doi: 10.1007/s00436-021-07384-5) it is stated “knockdown of one lncRNA in Schistosoma mansoni, SmLINC156349, which led to … pairing impairment, … and 33% reduction in female oviposition“. Both sentences in the text should be rephrased accordingly.*

Following the Reviewer recommendation, we rephrased the sentences on lines 25-26 and 99-103.

*Lines 142 ff (Selection of lincRNAs to be tested) Why did the authors particularly focus on the functional characterization of LINC RNAs, and not LNCA or LNCS RNAs? Was this decision based on the obtained RNAseq results? I.e. was it more likely to obtain phenotypes regarding pairing and reproduction when knocking down lincRNAs compared to the other groups? The focus on lincRNAs should be explained to the reader.*

The intergenic lncRNAs were chosen because interfering with their expression levels with knockdown approaches (such as using double-stranded RNAs) does not suffer from the problem of a simultaneous artifactual knockdown of a protein-coding message from the same locus. In fact, targeting a sense or an antisense lncRNA with double-stranded RNAs (dsRNAs) may unwantedly reduce the expression of the protein-coding gene that is expressed from the locus, because the dsRNAs will share a stretch of its sequence with an intron or exon of the immature pre-mRNA of the protein-coding gene message transcribed in the locus. An intergenic lncRNA is, by definition, away from the locus of a protein-coding gene, thus it will not share any stretch of sequence with the pre-mRNA message of a protein-coding gene.

A short explanation has now been added on lines 163-166 and a longer explanation on lines 132-141 in the Supplementary Methods in S1 Text.

*The reference list should be checked for consistency in the format of the individual references (species names should be written in italics; capitalization in article titles should be removed; etc.)*

As requested by the Reviewer we have checked the FONT format of the References list.

*Figures

Fig. 5 and Fig. 9: Please specify in the legend for which graph which statistical test was applied (t-test or ANOVA).*

As requested by the Reviewer, we have added to each legend in Fig. 5 and Fig. 9 the name of the statistical test used in the analyses of each panel (lines 1304-1305 and 1365-1366).

*Fig. 5D and E, and lines 230f: The degree of motility reduction does not match the degree of viability as measured by ATP levels. In fact knockdown of the two lncRNAs with least effects on motility led to the highest drop of ATP. How is this interpreted?*

Please note that lincRNA knockdown with dsLINC101519 (or dsLINC124324) caused the highest reduction in worms’ viability, as measured by reduced ATP levels (Fig. 5E), while showing a reduced egg cells proliferation (Fig. 5I) and a sustained female somatic stem cells proliferation (new Supplementary Figure 4 in S1 Text), as measured by EdU labelling. This suggests that, when parasite homeostasis was affected by knockdown of certain lincRNAs, egg and body cell proliferation might be the sources of highest ATP consumption, as opposed to worm motility. In fact, as the Reviewer pointed out, knockdown of dsLINC101519 caused the least effect on motility (Fig. 5D). On the other hand, motility was reduced by 60 to 90% upon knockdown of the other two lincRNAs (dsLINC110098 and dsLINC175062) which was not accompanied by reduction in ATP levels (Fig. 5E), again suggesting that the effects on motility were not directly correlated with ATP levels. We have now added this interpretation to the Discussion (lines 467-481).

*Fig. 5: It would be valuable to add some representative images of worms and eggs to the supplementary data, which depict the phenotype of the worms, the shape/size of eggs, the egg autofluorescence and EdU/DAPI staining of eggs - as a minimum from one control condition and one selected RNAi condition, to give the reader an idea how a significantly reduced parameter looks like.*

As requested by the Reviewer, we have now added Supplementary Figure 2 in S1 Text with representative images of eggs (lines 260-276 in S1 Text) and this Supplementary Figure is mentioned in the main text (lines 286-287). Please note that representative images of worms are already shown in Figs. 6 and 7.

*Fig. 8 and other ISH figures: Were sense probes used to check for unspecific signals in ISH? Representative images of worms treated with sense probes should be added to the supplementary information or main figure. This is needed, amongst others, to correctly interpret situations where the sc expression data (no signal) do not match the ISH results (strong signals), e.g. in Fig. 8D and suppl. Fig. 3B.*

Following the Reviewer’s recommendation, we have added negative control images of WISH with the sense probes for all lincRNAs in Supplementary Figures 6B and 8A to 8D in S1 Text (lines 371-381 and 397-405 in S1 Text). This is now described in the Methods in the main text (lines 879-882).

*Methods

The part “Parasite in vitro mimetic model of paired and unpaired adult worms“ reads like a mixed results and discussion chapter and is not fitting to a Materials & Methods chapter. Most of it should be transfered to the results and discussion part of the manuscript, or moved to the Supplementary Methods/Supplementary Data.*

Following the suggestion from the Reviewer, we have now moved part of this section to Results (lines 178 to 197) and another part to a new section, Supplementary Data in S1 Text (lines 23-85 in S1 Text). Essentially, we have placed a short version of testing the unpairing model with protein-coding genes under Results (lines 185-192 in the main text), and the full version of the results with protein-coding genes as Supplementary Data in S1 Text (lines 23-85 in S1 Text). We kept a short description of the procedures involved with the “**Parasite *in vitro* mimetic model of paired and unpaired adult worms”** in the Methods section (lines 638-653), while the results of protein-coding genes expression that were obtained as a control for characterization of the model and were originally in the Methods, have now been moved to the Results section (lines 185-192 in the main text) and to Supplementary Data in S1 Text (lines 23-85 in S1 Text), as described above.

*Line 608: (RNAi) “an in silico analysis of the transcript on-target and off-target bases was performed“ – describe how this was done, which software was used, and give a reference if possible. Was the probability of off-target knockdown of other (lnc)RNAs by the selected dsRNA sequences bioinformatically checked and excluded, e.g. by BLAST?*

As requested by the Reviewer, we have now added a complete description of the search procedure and the tool used (BLASTN against the transcriptome), with references to the transcriptome versions and to the tool used (lines 657-665, and 857-866). Yes, all selected lincRNA sequence segments matching any other transcript were excluded, and all probes that have been used do not match any other transcript, as we now describe in detail.

In fact, upon including this detailed information in the manuscript, since we had originally used the 2017 version of the *S. mansoni* protein-coding genes transcriptome [41], we decided to update the on/off-target analysis by searching against the recently published version 9 of the protein-coding genes transcriptome (23April2022) [48]. By doing this, we found that SmLINC154295 now maps to a region in the genome where a new protein-coding gene, Smp_348400.1 has been added in version 9 of the transcriptome (therefore SmLINC154295 is no longer intergenic but rather it is an antisense lncRNA, which overlaps to the last exon of Smp_348400.1, on the opposite strand), and therefore we have removed SmLINC154295 from the manuscript. We have also found that the segment of SmLINC124324 that had been used for dsRNA probe design has off-target matches to other transcripts in version 9, and we entirely removed SmLINC124324 from the manuscript.

*Line 630: Please give a reference for your selection of the mCherry gene to serve as non-related dsRNA control. Was it previously validated that mCherry dsRNA does not influence gene expression? Other typically used controls like GFP dsRNA unexpectedly have an influence, although not expressed in schistosomes.*

Yes, mCherry dsRNA has been previously tested and used in *S. mansoni* by the group of Dr. Conor R. Caffrey and we have now added the reference on lines 687-688.

*Line 642f: “At the end of the [RNAi] experiment, the adult worm couples were collected and stored in RNAlater“. Probably some other storage conditions need to be added to the text – or were EdU stainings and vitellaria stainings done on worms that were stored in RNAlater?*

We thank the Reviewer for noticing the lack of adequate description. We have now corrected the Methods, indicating that parasites were stored in RNAlater for RNA extraction, and were processed for microscope image acquisition when EdU labeling was performed (lines 700-703).

*Line 754: Indicate with which magnification powers the microscopic images were acquired, and which wavelengths were used for excitation of the four dyes.*

As requested by the Reviewer, we have now given these details on lines 820-828.

*Line 761: Specify which platform/tool was used (BLAST?) to search each lincRNA sequence against the previously published S. mansoni transcriptome*

As requested by the Reviewer, we have now indicated the tool used (BLASTN against the transcriptome) and we gave the search parameters (lines 657-665, and 857-866).

*A chapter describing the statistical test(s) is missing in the methods section.*

As requested by the Reviewer, we have added the topic “Statistical analyses” to the Methods (lines 883-891).

*Supplementary Methods in S1 Text: For transparency of the lincRNA selection pipeline, it would be appreciated to add to Appendix 2 Excel tables that list the genes that are highlighted with circles in Figures B-E and those indicated in Table B.*

As requested by the Reviewer, we have now indicated in the new Supplementary Table 24 in S2 Appendix the gene IDs for all lincRNA genes that are highlighted with circles in Figures B-G and those indicated in Table B. Upon fulfilling the Reviewer’s request, we noticed that there were a few missed steps in the selection pipeline described in Supplementary Methods in S1 Text. We have now added these steps to the Supplementary Methods along with new Figures F and G (lines 189-205 in S1 Text).

***Reviewer #3:***

*Line 98- the difference between lncRNA and lincRNA should be defined*

As requested by the Reviewer we have now added a description of intergenic lncRNAs (lincRNAs or SmLINCs), of antisense lncRNAs (SmLNCAs) and sense lncRNAs (SmLNCSs) to the Results on lines 137-142.
